# Role-specific household contact patterns in urban and rural China: Implications for influenza household transmission

**DOI:** 10.1016/j.idm.2026.09.001

**Published:** 2026-09-02

**Authors:** Tingting Guo, Bowen Ming, Yuxia Liang, Fayu Lu, Wenjing Ma, Juanjuan Zhang

**Affiliations:** a Shanghai Institute of Infectious Disease and Biosecurity, School of Public Health, Fudan University, Shanghai, People's Republic of China; b Key Laboratory of Public Health Safety, Ministry of Education, Fudan University, Shanghai, People's Republic of China

**Keywords:** Household contact patterns, Household structures, Family roles, Individual-based model, Vaccination

## Abstract

**Background:**

Household contact patterns significantly influence the household transmission dynamics of respiratory pathogens. Previous studies have shown ignoring the heterogeneity of household contact patterns can lead to an underestimation of the relative susceptibility and infectivity of children to influenza. However, there is a lack of studies on household contact patterns across all age groups and family structures in China.

**Methods:**

We conducted two cross-sectional studies via face-to-face, paper-based surveys in Anhua County, Hunan Province (June–July and October 2021) and Kunming City, Yunnan Province (February–April 2023). Factors associated with household contact patterns were explored using linear mixed-effects models. Role-specific contact matrices were established, stratified by household structures and crowding levels. Additionally, using influenza as a representative example, we developed a discrete-time, individual-based stochastic model to investigate household transmission dynamics initiated by different index case roles and to evaluate various role-targeted vaccination strategies.

**Results:**

The mean number of household contacts reported by participants was 2.58 (95% CI, 2.50–2.66) in Anhua County and 2.26 (95% CI, 2.19–2.33) in Kunming City. Household contact patterns were mainly associated with family roles, household structures, and the number of bedrooms. Contact duration was significantly longer between father–mother pairs, between mother–child pairs in two-generation households and mother–grandchild pairs in three-generation households, and among siblings. Mothers as index cases generated the highest number of secondary cases, whereas grandfathers caused the fewest. When grandparents or parents were the index cases, transmission most frequently occurred to children or grandchildren, whereas when children or grandchildren were the index cases, infection most commonly spread among siblings. Crowded living environments increased transmission risk, leading to a higher average number of secondary cases across all index case roles. Prioritizing vaccination of children or grandchildren yielded the greatest reduction in secondary cases, with further improvement when mothers were also vaccinated. Although vaccinating grandparents had minimal impact on reducing household transmission, it remained essential for their personal protection. Notably, vaccinating both grandparents and grandchildren together was the most efficient four-dose strategy for reducing infection risk for grandparents.

**Conclusions:**

Given the heterogeneity of household contact patterns, to reduce household secondary cases, priority should be given to vaccinating children or grandchildren. However, if the aim is to reduce the infection risk among grandparents, directly vaccinating them is more effective. Unlike traditional age-based vaccination strategies, our findings provide a new perspective to optimize vaccine allocation.

## 1 Introduction

Respiratory infectious diseases remain a leading contributor to the global burden of disease (Global and regional, 2024; Huang et al., 2022; Safiri et al., 2022). Numerous studies have demonstrated that households are significant settings for the transmission of respiratory infectious diseases (Madewell et al., 2020, 2021; Zhang et al., 2026). Evidence suggests once a household member contracts influenza, the infection risk for other household members can reach up to 38% (Tsang et al., 2016). The transmission of respiratory infectious diseases is mainly driven by social mixing patterns (Mossong et al., 2008; Zhang et al., 2020). A study from the Coronavirus Disease 2019 (COVID-19) pandemic revealed that although household contacts accounted for merely 6% of total social contacts, they were responsible for approximately 40% of all transmission events (Ferretti et al., 2024). Contact patterns among household members are non-random and more frequent, intense, and prolonged compared with community contacts (Goeyvaerts et al., 2018; Mousa et al., 2021). Research indicates that ignoring heterogeneous household contact patterns results in the underestimation of children's susceptibility and infectiousness parameters (Layan et al., 2024). Current studies focus on age-specific contact patterns and their impact on population transmission dynamics (Cencetti et al., 2021; Kiti et al., 2016; Sullivan et al., 2024). However, age-specific household contact patterns cannot fully explain some household transmission events. For instance, spouses exhibited a higher secondary attack rate (SAR) during the COVID-19 pandemic (Madewell et al., 2020); respiratory syncytial virus (RSV) household transmission mostly occurs from children to adults (Cox et al., 2026); and pertussis is more likely to spread among siblings (Lin et al., 2022). Furthermore, multigenerational living significantly elevates the risk of COVID-19 infection, severe illness, and mortality among older adults (Nafilyan et al., 2021; Stokes et al., 2020; Wing et al., 2022). Household structure and composition also influence infectious disease transmission (Endo et al., 2019; Møgelmose et al., 2023; Pellis et al., 2020). China has a large number of three-generation households where grandparents participate in childcare, leading to differences in parental childcare involvement between two-generation and three-generation households (Bai et al., 2024; Ko & Hank, 2013; Song et al., 2025; Zhou et al., 2021). Crucially, heterogeneity in household contact is significantly influenced by the division of roles within the household. Women are extensively involved in childcare and domestic labor (Cao et al., 2024; Chen, 2024; Chen et al., 2011; Liao et al., 2023; Zou et al., 2019).

However, few studies have focused on role-specific household contact patterns by structure and their impact on the household transmission of respiratory infectious diseases. Additionally, social mixing patterns differ between urban and rural settings (Read et al., 2014; Zhang et al., 2019). To address these limitations, we characterized role-specific household contact patterns by household structure in Anhua County and Kunming City, China, explored the factors associated with household contact patterns, and developed a discrete-time, individual-based stochastic SEIR model. This model was used to explore the impact of empirical household contact patterns on the household transmission of influenza and to assess the efficacy of role-specific vaccination strategies.

## 2 Methods

### 2.1 Study site, population and data collection

We conducted two cross-sectional studies at two sites in China. One was in Anhua County, Hunan Province, from June to July and in October 2021, and the other was in Kunming City, Yunnan Province, from February to April 2023. All participants were recruited from communities and schools. Households were randomly selected within each community, and some or all household members participated in the survey. Students were recruited through schools (Liang et al., 2024). Household members were defined as individuals who shared accommodation and meals for at least four days per week over the preceding three months. Participants completed paper-based questionnaires via face-to-face interviews to provide information on demographic characteristics, household composition, and social contacts on the day preceding the survey. A contact was defined as either a face-to-face conversation involving three or more words or direct skin-to-skin physical contact(Mossong et al., 2008). For each household and non-household contact, data were collected on the relationship to the participant, contact duration, co-presence duration, contact type (physical/non-physical contact), day type (weekday/weekend), and the demographic characteristics of the contacted individual.

### 2.2 Classification of household structure and data pre-processing

Only households with at least two members were included in the analysis. Household structures and individual roles were defined based on generational composition. Specifically, one-generation households consisted of a couple (with roles designated as father and mother). Two-generation households comprised one or both parents living with one or more children (with roles defined as father, mother, and child). Three-generation households included grandparents (roles specified as grandfather, grandmother, father, mother, and grandchild). All other household compositions or secondary roles (e.g., aunts, uncles, nieces, and nephews) were collectively classified as “other”.

Data collection for certain variables differed between the two sites. In Anhua, contact duration was recorded as a categorical variable (<5 min, 5–14 min, 15–59 min, 1–4 h, and >4 h). These categories were converted into numeric values using the midpoints of the first four intervals (2.5 min, 9.5 min, 37 min, and 2.5 h, respectively), and the highest category (>4 h) was censored at 4 h(Fu et al., 2012; Leung et al., 2017, 2023; Liang et al., 2024; Nelson et al., 2025). Contact duration in Kunming and co-presence duration at both sites were measured as continuous variables.

### 2.3 Statistical analysis

#### 2.3.1 Descriptive analysis

All analyses were performed separately for Anhua and Kunming. We calculated five summary indicators for each participant: number of contacts, total contact duration (calculated as the sum of all household contact duration), total co-presence duration, per capita contact duration, and per capita co-presence duration. The per capita indicators were derived by dividing the respective totals by the total number of household contacts. Descriptive statistics included means with two-sided 95% confidence intervals (CIs) estimated using a nonparametric bootstrap (percentile method with 1000 resamples), as well as medians with interquartile ranges (IQRs). Analyses were stratified across four categories of explanatory variables: (i) sociodemographic characteristics, including sex, age group, education level, occupation, and total family income (in Chinese Yuan, CNY); (ii) characteristics of the contact diary day, including day type (i.e., weekday or weekend), whether the day was typical, and weather conditions (i.e., sunny, cloudy, rainy, or varied); (iii) household characteristics, comprising household structure (i.e., one-, two-, and three-generation households or other), family role, number of bedrooms in use, and household size; and (iv) other participant information, specifically whether the participant had an underlying disease and the self-rated accuracy of contact recall (i.e., very well, well, moderately well, or not well). Notably, data concerning the number of bedrooms in use and weather conditions were exclusively collected in Anhua.

#### 2.3.2 Factors associated with household contact duration and co-presence duration

To identify factors associated with household contact patterns in one-, two-, and three-generation households, we employed linear mixed-effects models for four summary indicators: total contact duration, total co-presence duration, per capita contact duration, and per capita co-presence duration. Given the right-skewed distribution and the presence of zero-valued observations, all outcome variables were transformed using log(Y+1). Models were fitted separately for Anhua and Kunming as follows:

(1) $$\begin{array}{c} \log\left(\;Y_{ik}+1\right)=\beta_{0}+f\left(Age_{ik}\right)+\sum_{p}\beta_{p}X_{p,ik}+u_{k}+\varepsilon_{ik\;} \end{array}$$

$$u_{k}∼N\left(0,\sigma_{u}^{2}\right),\varepsilon_{ik}∼N\left(0,\sigma^{2}\right)$$

where $Y_{ik}$ is the response variable for the participant i in the household k, and $\beta_{0}$ is the fixed intercept. The term $f\left(Age_{ik}\right)$ denotes a potentially nonlinear effect of age, modeled using a natural spline with 4 degrees of freedom. $X_{p,ik}$ represents the p-th fixed-effect predictor (e.g., household structure, family role, occupation) for the participant i in household k, with $\beta_{p}$ being the corresponding regression coefficient. $u_{k}$ is a household-level random intercept accounting for within-household clustering and is assumed to follow a normal distribution, $u_{k}∼N\left(0,\sigma_{u}^{2}\right)$ . $\varepsilon_{ik}$ is the residual error term, also assumed to follow a normal distribution, $\varepsilon_{ik}∼N\left(0,\sigma^{2}\right)$. We calculated the point estimates and corresponding 95% confidence intervals (CIs) for each explanatory variable.

To select explanatory variables for the final models, we assessed potential collinearity among categorical predictors using Cramér's V statistic. We identified strong associations between family role and sex as well as between household structure and household size. Consequently, we excluded sex and constructed a new categorical variable, 'standardized household size', which categorizes the deviation of household size from the structure-specific mean household size (rounded to the nearest integer). These categories are defined as: two or more fewer persons (≤−2), one fewer person (−1), the baseline (0), one additional person (+1), and two or more additional persons (≥+2) relative to the structure-specific mean. Finally, we fitted the linear mixed-effects model defined above, including the following variables: household structure, standardized household size, family role, occupation, education, family income, underlying disease, day type, recall accuracy, typical day, number of bedrooms, and weather. All predictors in the final models had a degrees-of-freedom–adjusted generalized variance inflation factor $GVIF^{\frac{1}{2df}}$ below 2, indicating no evidence of multicollinearity.

#### 2.3.3 Construction of household contact duration and contact co-presence matrix

To quantify contact patterns across different household structures, we constructed symmetric, role-specific, and age-specific matrices for both contact duration and co-presence duration, restricting the analysis to core roles (excluding “other” roles). Treating contact pairs as undirected, we first calculated the mean contact (or co-presence) duration, denoted as $d_{h,ij}$ for each specific pair (i, j) within household h. Subsequently, we employed a household-unit bootstrap resampling method with 1000 iterations. For each bootstrap iteration b, the mean duration for pair (i, j), denoted as $m_{ij}^{\left(b\right)}$, is defined as:

(2) $$\begin{array}{c} m_{ij}^{\left(b\right)}=\frac{1}{N_{ij}^{\left(b\right)}}\sum_{h\in S_{b}}\;d_{h,ij}\; \end{array}$$

where $S_{b}$ is the set of households sampled in the *b*-th bootstrap iteration, and $N_{ij}^{\left(b\right)}$ is the number of households sampled in the *b*-th iteration that contain the (i, j) pair. The final matrix values were derived from the mean of the bootstrap distribution, with 95% *CI*s calculated using the 2.5th and 97.5th percentiles. We stratified the contact matrices by household structure for both study sites. To further investigate the effect of housing density in Anhua, we performed an additional stratification based on the number of bedrooms per capita (calculated as the number of bedrooms divided by household size). Based on the sample median of 0.6 bedrooms per capita as the threshold, households were classified as either “crowded” (<0.6) or “not crowded” (≥0.6).

#### 2.3.4 Within-household transmission model

##### 2.3.4.1 Within-household influenza-like infection transmission model

We simulated within-household influenza transmission using a stochastic, discrete-time, individual-based model with daily time steps. Each individual's state could be susceptible (S), exposed (E), infectious (I), or removed (R).

Two common household compositions were considered: (i) two-generation households consisting of a father, a mother, and two children; and (ii) three-generation households consisting of a grandfather, a grandmother, a father, a mother, and two grandchildren.

Each individual occupied one of four epidemiological states: S, E, I, or R. The latent and infectious periods were fixed at 2 and 5 days, respectively (Carrat et al., 2008). Each simulation was run for up to 21 days (Rudge et al., 2019; Simmerman et al., 2011) and terminated early if no exposed or infectious individuals remained. Within each defined scenario (stratified by location and household structure), every household member was assigned as the index case sequentially, with 10,000 independent Monte Carlo simulations conducted for each index case (i.e., 10,000 simulations per index case within each scenario). Additionally, scenarios in Anhua were further stratified by housing density (crowded versus spacious). Let $C_{i,j}$ denote the daily contact duration between household members *i* and j (hours/day), derived from our empirical matrices. On day t, each susceptible individual (j) could be infected by any infectious household member i∈I(t), where I(t) denotes the set of infectious individuals on that day. We assumed independent competing infection hazards within the same day. The daily probability of transmission from an infectious individual *i* to a susceptible individual j was defined as:

(3) $$\begin{array}{c} P_{i,j}\left(t\right)=1-\exp\left(-\beta \cdot C_{i,j}\cdot \mu_{i}\cdot \sigma_{j}\right) \end{array}$$

where *β* is the transmission coefficient, *μ*_*i*_ represents the relative infectivity of role *i*, and $\sigma_{j}$ represents the relative susceptibility of role j. Relative susceptibility was set to 2.0 for children/grandchildren and 1.0 for adults, while relative infectivity was set to 1.0 for all roles (Layan et al., 2024). Under the independent-hazard assumption, the probability that susceptible individual j becomes infected on day *t* was calculated as:

(4) $$\begin{array}{c} P_{\left(\inf,j\right)}\left(t\right)=1-\prod_{i\in I\left(t\right)}\left(1-P_{i,j}\left(t\right)\right)\; \end{array}$$

Following the introduction of the index case, transmission was assumed to occur exclusively within the household, meaning that between-household transmission was not considered.

##### 2.3.4.2 Transmission chain reconstruction

To construct the transmission chain in the simulation, when individual *j* becomes infected on day *t* and there are multiple sources of infection, this study employed a continuous-time competing-risk model to identify the source of infection within a single time step. The final source of infection was determined through probability sampling (the probability of assigning source *k* to individual *j* is proportional to the instantaneous infection risk posed by that source):

(5) $$\begin{array}{c} P\left(\text{infector}=k|j\;\text{infected}\;\text{at}\;t\right)=\frac{-\ln\left(1-P_{k,j}\left(t\right)\right)}{\sum_{i\in I_{t}}-\ln\left(1-P_{i,j}\left(t\right)\right)} \end{array}$$

Infector–infectee counts were aggregated across simulations to obtain empirical transmission pathway frequencies and role-specific transmission probabilities.

##### 2.3.4.3 Calibration of transmission coefficient β

For each location, the transmission coefficient was calibrated using the cumulative infection probability over the infectious period for the father-to-mother dyad: $\begin{array}{c} 1-\exp\left(-\beta \;C_{FM}\;D_{\inf}\right)={SAR}_{FM} \end{array}$

(6) $$\begin{array}{c} \beta =\frac{-\ln\left(1-{SAR}_{FM}\right)\;}{C_{FM}\cdot D_{\inf}} \end{array}$$

where $C_{FM}$ represented the location-specific average contact duration between a father and a mother, calculated as the arithmetic mean of the contact duration in both Two-Generation and Three-Generation household contact duration matrices, and $D_{\inf}$ was the infectious duration, and ${\;\text{SAR}}_{\text{FM}}$ was the secondary attack rate between father and mother. We selected the father–mother dyad because their relative susceptibility and infectivity were set to 1. Consequently, calibration to this dyad was not influenced by the relative susceptibility of children and grandchildren. In addition, the father–mother dyad was present in both modeled household structures, thereby providing a reference across the two-generation and three-generation households. Importantly, both the calibration framework and the value of ${SAR}_{FM}=13\%$ were adopted from the influenza-like simulation parameterization used in a previous study(Layan et al., 2024).

##### 2.3.4.4 Outcome measures

For the household transmission model with a pre-specified index case, the primary outcome was the number of secondary cases (K) within the household. To elucidate transmission mechanisms, we additionally reconstructed transmission chains and estimated role-specific transmission probabilities (e.g., mother-to-child transmission), which were summarized as directed transmission networks.

#### 2.3.5 Vaccination intervention modeling

##### 2.3.5.1 Weighted index-case framework with heterogeneous external introduction risk

Vaccination was modeled as a leaky protection mechanism that reduces susceptibility. We assumed the vaccine effectiveness against susceptibility ($VE_{S}$ = 0.71) and infectiousness ($VE_{I}$ = 0) (Tsang et al., 2019). For a vaccinated individual, the susceptibility $VE_{S}$ and infectivity $VE_{I}$ parameters were adjusted as follows:

(7) $$\begin{array}{c} \mu_{i}^{vac}=\left(1-VE_{I}\right)\;\mu_{i\;} \end{array}$$

$$\sigma_{j}^{vac}=\sigma_{j}\left(1-VE_{S}\right)$$

Accordingly, the daily probability $P_{i,j}$ of a susceptible individual j being infected by an infectious individual i at time t is given by:

(8) $$\begin{array}{c} P_{i,j}\left(t\right)=1-\exp\left(-\beta \cdot C_{i,j}\cdot \sigma_{j}^{vac}\cdot \mu_{i}^{vac}\right) \end{array}$$

Vaccination strategies were defined by the number of available doses and their allocation across different combinations of household members according to predefined prioritization rules. For each index case *i* and vaccination strategy *V* in the household, we performed 10,000 stochastic Monte Carlo simulations to record the distribution of secondary cases within the household.

To account for varying levels of community exposure among different family roles, we evaluated vaccination strategies using a weighted index-case framework adapted from Prunas et al. (Prunas et al., 2022). The risk of external infection for member i, denoted as $P_{ext,i}$, represents the probability of becoming the index case from outside the household. Based on published data, we assigned role-specific external risks: 18% for children/grandchildren, 3.8% for fathers, 3.5% for mothers, and 1.3% for grandparents (Endo et al., 2019). These external risks were used exclusively to determine the probability distribution of the index case. In addition, the external risks did not contribute to the force of infection during within-household transmission. Under a given strategy *V*, the unnormalized introduction weight ($W_{i}$) for member i was defined as:

(9) $$\begin{array}{c} W_{i}=P_{ext,i}\cdot \left(1-v_{i}\cdot VE_{S}\right) \end{array}$$

where $v_{i}$ is a binary indicator of vaccination status (1 if vaccinated, 0 otherwise).

##### 2.3.5.2 An assessment aimed at reducing the number of secondary cases in the home

Vaccination coverage was defined based on the number and specific roles of the vaccinated members. We evaluated vaccination strategies in which from one to all household members were vaccinated, considering different role combinations. For each vaccination strategy V, the number of secondary cases within a household (AS) was calculated as:

(10) $$\begin{array}{c} AS\left(V\right)=\sum_{i\in Household}\left(\frac{W_{i}\left(V\right)}{\sum_{k\in Household}W_{k}\left(V_{baseline}\right)}\right)\cdot SC\left(i,V\right) \end{array}$$

Here, SC(i,V) represents the average number of secondary cases obtained from 10,000 simulations under vaccination strategy V, with *i* as the index case; $W_{i}\left(V\right)$ represents the weight of virus introduction by individual *i* under strategy *V*. The number of secondary cases in the unvaccinated (baseline) scenario was calculated as:

(11) $$\begin{array}{c} AS\left(V_{baseline}\right)=\sum_{i\in Household}\left(\frac{P_{ext,i}}{\sum_{k\in Household}P_{ext,k}}\right)\cdot SC\left(i,\text{unvax}\right) \end{array}$$

In this study, vaccination effectiveness is defined as the percentage reduction in the number of secondary cases within households under each vaccination strategy:

(12) $$\begin{array}{c} VE\left(V\right)=\left(1-\frac{AS\left(V\right)}{AS\left(V_{baseline}\right)}\right)\times 100\% \end{array}$$

The 95% confidence interval was obtained through 1000 bootstrap resampling runs, in which the final infection levels for the unvaccinated group and under each strategy were resampled with replacement, and the number of secondary cases and the percentage reduction were recalculated.

##### 2.3.5.3 An assessment aimed at reducing the risk of infection among grandparents

For three-generation households, this study additionally assessed the impact of different vaccination strategies on the risk of infection among grandparents. The average probability of infection for both grandparents across all possible initial cases was calculated as:

(13) $$\begin{array}{c} P\left({GP}_{\text{infected}}|V\right)=\sum_{i\in \text{Household}}\frac{W_{i}\left(V\right)}{\sum_{k}W_{k}\left(V_{\text{baseline}}\right)}\cdot \frac{P\left(\text{GF}\;\text{infected}|i\right)+P\left(\text{GM}\;\text{infected}|i\right)}{2} \end{array}$$

where P(GFinfected∣i) and P(GMinfected∣i) denote the probabilities that the grandparents of the index case i are infected. GP is the abbreviation for grandparents. For each combination of strategy V and index case *i*, we calculated the point estimate $\hat{p}\left(i,V\right)$ of the average infection probability of grandparents from 10,000 simulations and quantify its uncertainty using a Beta–binomial conjugate model. Let $\hat{p}\left(i,V\right)$ be the point estimate of the infection probability, and assume that $k=\text{round}\left(\hat{p}\times 1000\right)$ infection events are observed in n = 1000 independent trials. Then, the posterior distribution of the infection probability was:

(14) $$\begin{array}{c} p\left(i,V\right)∼\text{Beta}\left(k+0.1,n-k+0.1\right) \end{array}$$

Here, 0.1 corresponds to a weak, approximately uniform prior. In each bootstrap iteration, independent samples were drawn from the posterior distributions corresponding to each index case, and then weighted and aggregated according to Equation (13) to obtain the posterior distribution of the risk of infection among grandparents under Strategy V (5000 iterations in total). The results were summarized using the median and interquartile range (IQR) of this distribution.

All analyses described above were performed using R version 4.3.2 for data cleaning, analysis, and visualization.

#### 2.3.6 Sensitivity analysis

To evaluate the robustness of the role-specific transmission patterns and vaccination-priority strategies, we considered in our sensitivity analysis: (i) the truncation value of “>4 h” contact duration, (ii) relative susceptibility of children/grandchildren (from 1.00 to 3.00 in increments of 0.25), (iii) thresholds used to classify households as crowded or non-crowded (0.50, the sample 60th percentile of 0.67, and 0.70 rooms per capita), (iv) SAR of the father–mother pair (5%, 8%, 20%, and 30%), (v) role-specific external infection risks, (vi) the latent period (from 1 to 3 days), (vii) the infectious period (from 3 to 7 days), (viii) vaccine efficacy against susceptibility (40% to 90%), and (ix) vaccine efficacy against infectiousness (15% to 60%).

For truncation values, we set the truncation values for the >4 h category in Anhua to 5, 6, and 8 h, respectively. In addition, the empirical contact-duration records exceeding 4 h in the Kunming data were truncated to 4, 5, 6, and 8 h to assess whether the results were sensitive to the truncation value.

We conducted three sensitivity analyses of role-specific external infection risks: (1) The external infection risks of the grandfather and grandmother were increased from 1- to 10-fold their main analysis values, while the risks of the other roles were held constant; (2) The grandchild risk was varied from 0.25- to 5-fold its main analysis value, while the risks of both grandparents were varied simultaneously from 1- to 10-fold their respective main analysis values; (3) We analyzed 5000 scenarios by independently sampling the external infection risk of each household role from a range of 0.5- to 2-fold its corresponding main analysis value.

## 3 Results

### 3.1 Description of study population and household contact patterns

This study included 1227 eligible participants from 890 households in Anhua County. Among these, 884 households were classified as one-, two-, or three-generation households, and 6 households were classified as other categories. The average size of three-generation households in Anhua was 4.62 individuals, with a mean of 1.50 participants per household. In Kunming, 985 participants from 780 households were enrolled, of which 774 were categorized as one-, two-, or three-generation families. Three-generation households in Kunming had a mean size of 4.73 individuals, with an average of 1.36 participants per household (Fig. S1).

Participants in Anhua and Kunming reported a mean of 2.58 (95% CI: 2.50–2.66) and 2.26 (95% CI: 2.19–2.33) household contacts, respectively. In Anhua, total contact duration, per capita contact duration, total co-presence duration, and per capita co-presence duration were 5.91 (95% CI: 5.69–6.15), 2.27 (95% CI: 2.20–2.35), 25.43 (95% CI: 24.43–26.48), and 10.09 (95% CI: 9.73–10.45) hours, respectively; for Kunming, the four corresponding durations were 7.77 (95% CI: 7.23–8.33), 3.54 (95% CI: 3.30–3.78), 16.75 (95% CI: 15.85–17.63), and 7.76 (95% CI: 7.40–8.11) hours, respectively (Table S1). The medians and IQRs for these summary indicators are shown in Table S2.

Total contact duration, per capita contact duration, total co-presence duration, and per capita co-presence duration all exhibited right-skewed distribution (Fig. S2). In both Anhua and Kunming, total contact and co-presence duration were highest in three-generation households, whereas per capita contact and co-presence duration peaked in one-generation households. In terms of specific roles in Anhua, fathers in two-generation households reported higher per capita contact and co-presence durations than their counterparts in three-generation households. Moreover, all four duration metrics for grandchildren in three-generation households exceeded those for children in two-generation households (Fig. 1)*.*

**Fig. 1:**
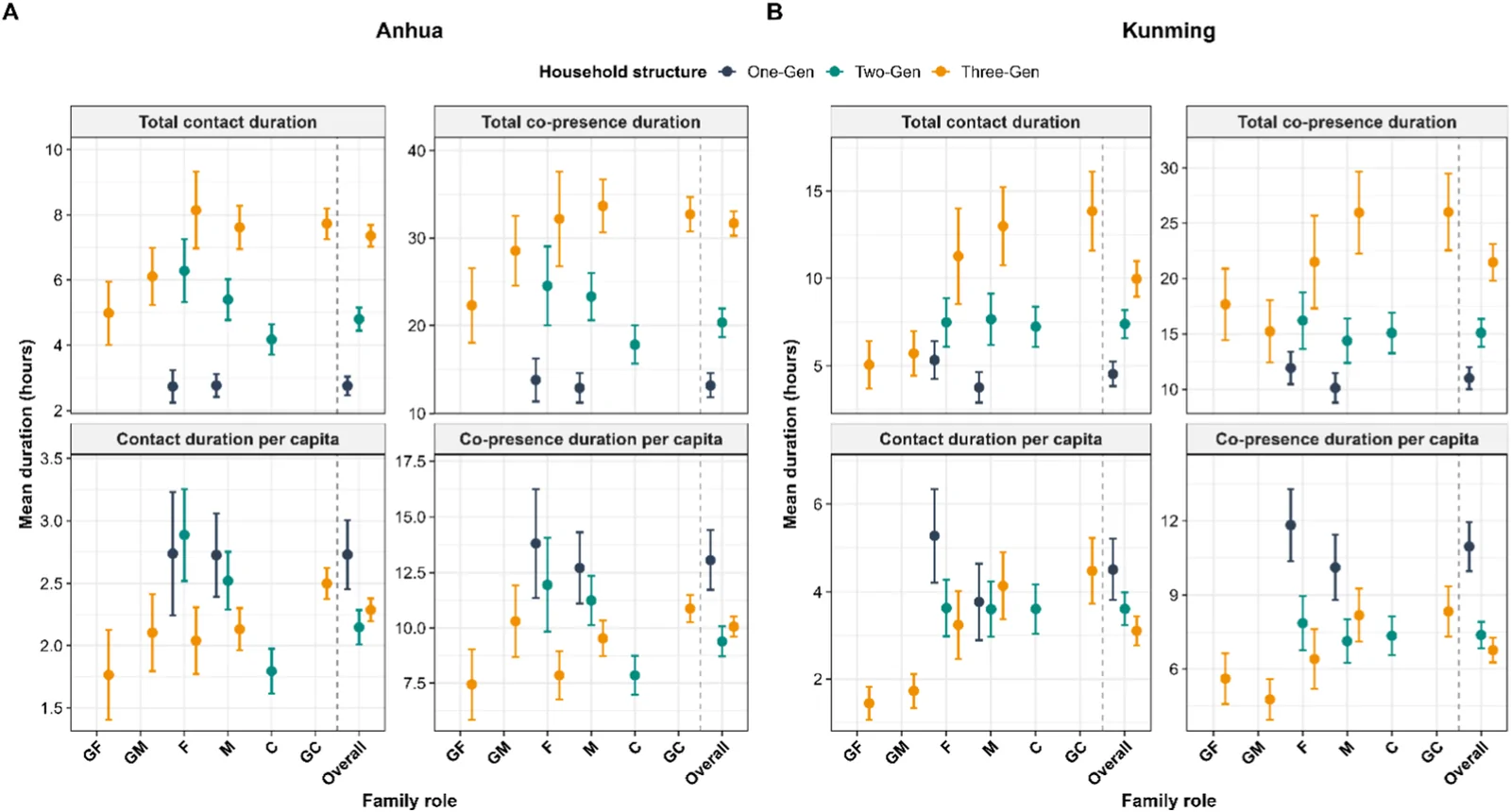
Four duration metrics by household structures and family roles. Point plots showing mean contact duration (hours) and 95% confidence intervals by family role and household structure in Anhua **(A)** and Kunming **(B)**. Abbreviations on the X-axis denote specific roles: GF, grandfather; GM, grandmother; F, father; M, mother; C, child; and GC, grandchild. Colors indicate the household structure type: blue for one-generation (One-Gen), green for two-generation (Two-Gen), and orange for three-generation (Three-Gen). The dashed vertical line separates specific family roles from the overall average.

### 3.2 Factors associated with contact duration and co-presence duration

Fig. S3 illustrates that total contact and co-presence duration were significantly longer in three-generation households than in one-generation households in both Anhua and Kunming. However, the association between household structure and per capita metrics differed by location. In Anhua, individuals in two- and three-generation households recorded significantly higher per capita contact and co-presence duration compared with those in one-generation households. In contrast, in Kunming, per capita contact and co-presence duration were highest in one-generation households. In Anhua, standardized household size was positively associated with all four metrics. However, in Kunming, no significant associations were observed for per capita metrics. Across both sites, children and grandchildren consistently exhibited significantly lower values for all four metrics compared with fathers. In Anhua, all four metrics decreased significantly as the number of bedrooms increased, and participants with underlying health conditions reported significantly lower per capita contact and co-presence duration than those without such conditions. Both total and per capita co-presence duration were higher on weekends in Anhua, whereas in Kunming, all four duration metrics were significantly higher on weekends (Fig. 2).

**Fig. 2:**
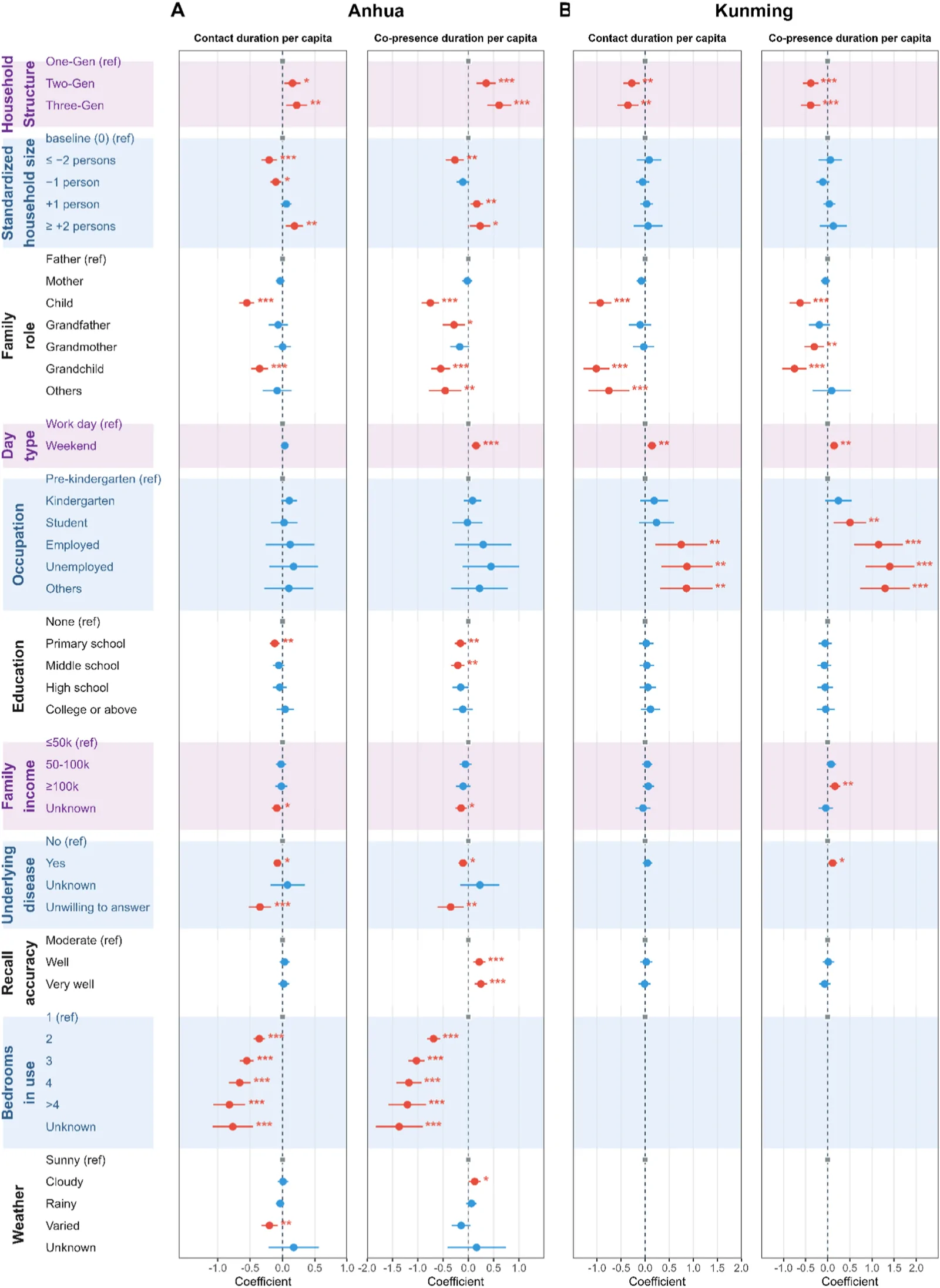
Forest plots of per capita contact and co-presence duration in Anhua and Kunming. Panel **(A)** displays the results for Anhua, and Panel **(B)** displays the results for Kunming. Points represent the regression coefficients (β), and horizontal error bars indicate the 95% confidence intervals (CIs). Red markers and lines denote statistically significant associations, whereas blue indicates non-significant results. Gray squares represent the reference categories. Asterisks indicate the levels of statistical significance (∗*p* < 0.05, ∗∗*p* < 0.01, ∗∗∗*p* < 0.001).

### 3.3 Contact duration matrix and co-presence duration matrix

In Anhua, among one-generation households, individuals aged ≥60 years reported the longest co-presence duration (15.65, 95% CI: 12.74–18.45) (Fig. S4A). In two-generation households, father-mother pairs recorded the longest contact duration (2.90, 95% CI: 2.57–3.21) and co-presence duration (11.74, 95% CI: 10.13–13.31), followed by child-child pairs, and then mother-child pairs; contact duration of father-child pairs was the shortest. In three-generation households, contact duration between father-mother pairs remained the longest (3.22, 95% CI: 2.90–3.53), followed by grandchild-grandchild pairs and mother-grandchild pairs (which exceeded father-grandchild interaction). Grandparent-parent interaction was the shortest. Notably, co-presence duration for mother-grandchild pairs was the longest overall (13.87, 95% CI: 12.90–14.94) (Fig. 3A).

**Fig. 3:**
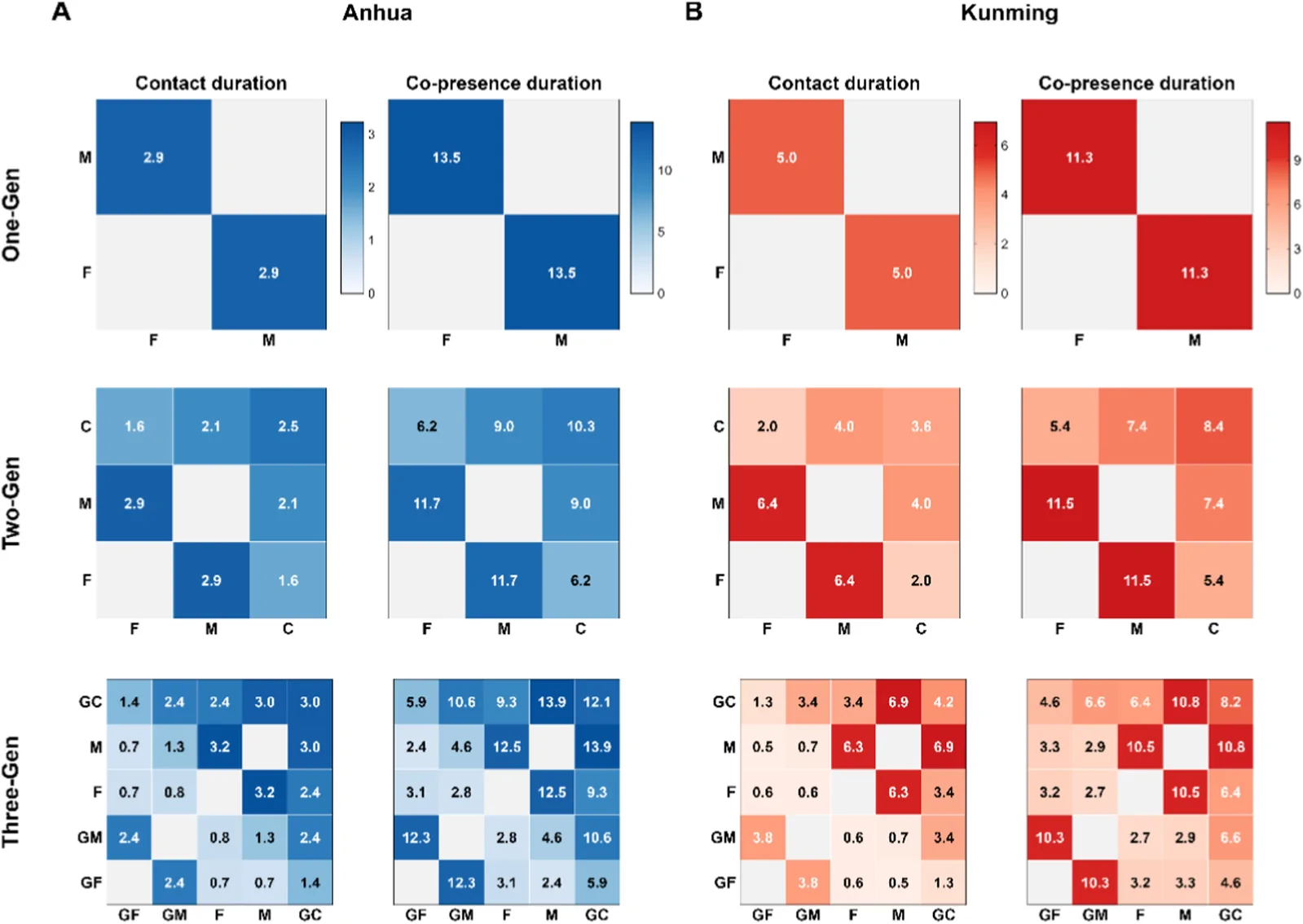
Role-specific contact and co-presence duration matrix across different generational structures in Anhua and Kunming. **(A, B)** Heatmaps display the mean duration of contact and co-presence between distinct family roles (GF: grandfather, GM: grandmother, F: father, M: mother, C: child, GC: grandchild) within one-generation (One-Gen), two-generation (Two-Gen), and three-generation (Three-Gen) households in Anhua **(A)** and Kunming **(B)**. Numeric values inside the cells represent the mean duration in hours, derived from 1000 bootstrap resamplings. Color gradients denote the length of time, with darker shades corresponding to longer duration.

Role-specific matrix in Kunming was broadly similar, with one key distinction: in three-generation households, contact duration between mother-grandchild pairs was the longest (6.92, 95% CI: 5.81–8.19), followed by father-mother pairs (Fig. 3B); Age-specific matrix revealed that, within three-generation households, the longest contact duration occurred in interactions between individuals aged 20–59 and 0–19 years (Fig. S4B).

In Anhua, household crowding substantially increased both contact and co-presence duration. In two-generation households, the largest difference was observed in father-child pairs, with their contact duration increasing nearly threefold under crowded conditions. In three-generation households, most pairs displayed higher duration under crowded conditions, with the exception of the relatively stable father-mother pairs (Fig. S5). Age-stratified matrix also confirmed that crowded households reported higher contact and co-presence duration (Fig. S6).

### 3.4 Results of household transmission model and vaccine scenarios

#### 3.4.1 Household transmission by different household structures and index roles

In Anhua, mothers as index cases exhibited the lowest probability of generating zero secondary cases in both two-generation (60.11% [95% CI: 59.15–61.07%]) and three-generation households (46.00% [95% CI: 45.02–46.98%]), whereas grandfathers showed the highest in three-generation households (65.41% [95% CI: 64.47–66.34%]) (Fig. 4A). Correspondingly, mothers generated the highest average number of secondary cases among all household members, with mean values of 0.59 (95% CI: 0.57–0.60) and 1.04 (95% CI: 1.01–1.06) in two- and three-generation households, respectively, while grandfathers as index cases yielded the lowest average (0.68 [95% CI: 0.66–0.70]). Similar trends were observed in Kunming (Fig. 4B). The sensitivity analysis of truncation, father-mother pair, latent period and infectious period showed mothers consistently remained the top-ranked index role (Fig. S7–S10). The susceptibility sensitivity analysis results showed the only exception regarding the mother's ranking occurred in Anhua three-generation households when the relative susceptibility of grandchild was reduced to 1.0, where the grandchild ranked first, and the mother ranked third (Fig. S11).

**Fig. 4:**
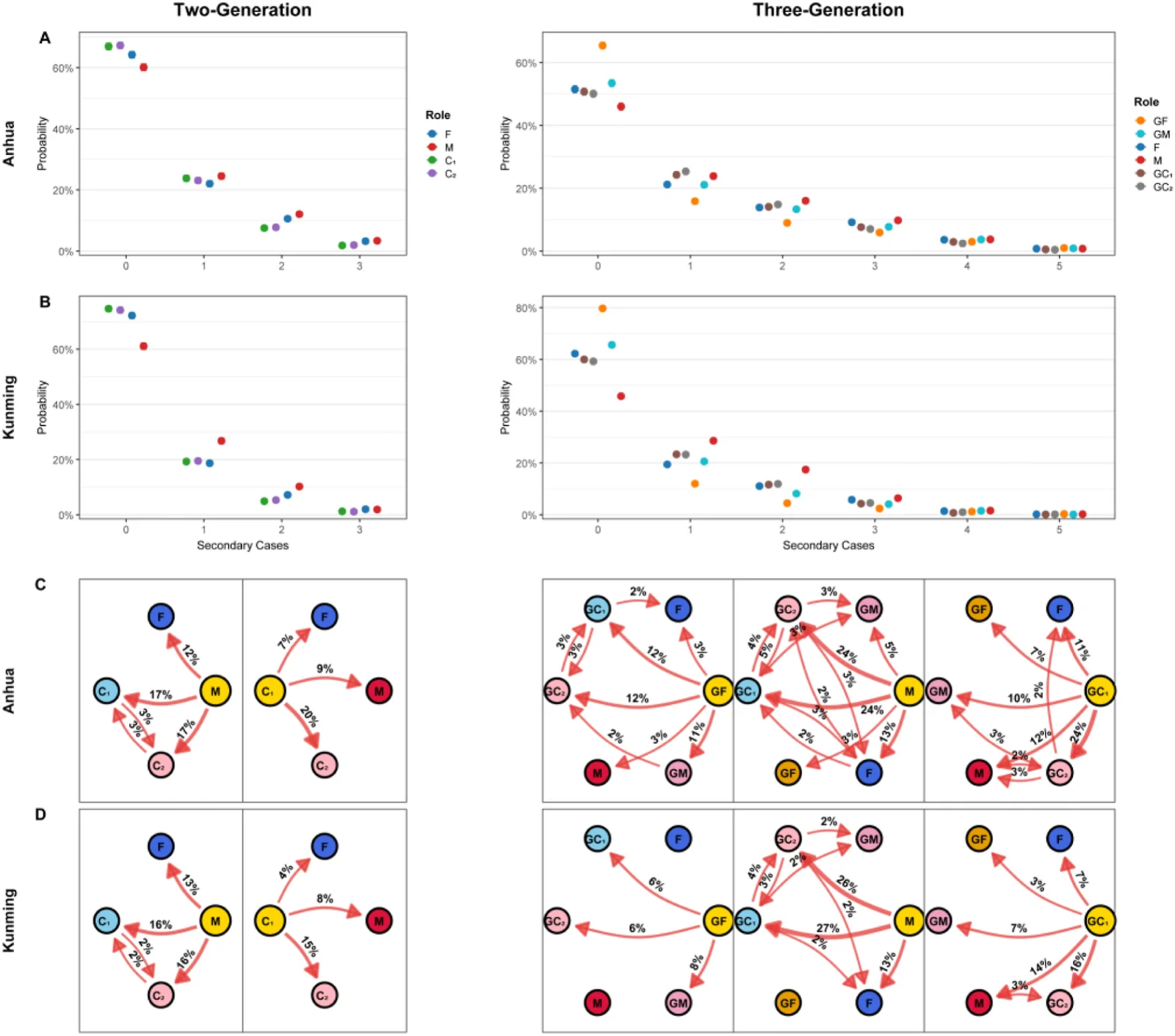
Role-specific secondary case distribution and transmission chains across different household structures in Anhua and Kunming. **(A, B)** The probability distribution of the number of secondary cases generated by different index case roles in two-generation and three-generation households in Anhua **(A)** and Kunming **(B)**. Points represent the mean probabilities derived from 10,000 stochastic SEIR model simulations, and error bars indicate the 95% confidence intervals. **(C, D)** Directed chains illustrating the within-household transmission pathways initiated by selected index cases in Anhua **(C)** and Kunming **(D)**. The index case is highlighted with a larger, gold-colored node. Nodes represent individual household members (GF: grandfather, GM: grandmother, F: father, M: mother, C_1_/C_2_: children, GC_1_/GC_2_: grandchildren). Edge thickness and corresponding percentage labels denote the transmission probability.

In Anhua, household crowding elevated the transmission risk for all roles, with fathers in two-generation households experiencing the most substantial decrease in the probability of generating zero secondary cases (from 76.85% [95% CI: 76.01–77.67%] to 56.18% [95% CI: 55.21–57.15%]) (Fig. S12). Sensitivity analyses using alternative household crowding thresholds showed that the mean number of secondary cases generated by each index role, as well as the mean across household roles, was higher in crowded households than in non-crowded households (Figs. S13 and S14).

Analysis of transmission chains in Anhua revealed that when a parent or grandparent was the index case, the infection was most frequently transmitted to children or grandchildren (Fig. 4C, Fig. S15). Conversely, when a child or grandchild was the index case, transmission most commonly occurred among siblings (with probabilities of 20.45% and 24.04% in two- and three-generation households, respectively). In Kunming, the transmission pattern was largely consistent with that in Anhua, with one notable exception: in two-generation households, fathers were more likely to infect their spouses than their children (Fig. 4D, Fig. S15).

Source attribution analysis for susceptible roles showed that in Anhua, spouses served as the primary infection source for one another, and siblings were the leading source for each other in two-generation households. In three-generation households, the grandchild emerged as the predominant infection source for both parents and siblings; grandmothers were principally infected by grandchildren, whereas grandfathers were most frequently infected by grandmothers (Fig. S16A). In Kunming, however, mothers were the primary infection source for fathers in three-generation households. Notably, mothers were the leading source for the child as well as the grandchild (Fig. S16B)*.*

#### 3.4.2 Results of different vaccination strategies

In Anhua County, under highly constrained vaccine supply (one dose per household), prioritizing a single child or grandchild in two-generation and three-generation households reduced secondary cases by 49.7% (95% CI: 48.2%–51.2%) and 45.4% (95% CI: 44.3%–46.6%), respectively. Notably, these single-dose strategies outperformed the vaccination of both parents (two-generation: 44.9%, 95% CI: 43.2%–46.7%; three-generation: 39.4%, 95% CI: 38.1%–40.9%) or both grandparents (24.8%, 95% CI: 23.2%–26.4%). When supply allowed for two doses, the optimal strategy for two- and three-generation households was to vaccinate two children (77.5%, 95% CI: 76.8%–78.2%) or two grandchildren (73.5%, 95% CI: 73.0%–74.1%), respectively. With three available doses, adding maternal vaccination to the two children or grandchildren further increased the reduction in secondary cases to 86.7% (95% CI: 86.3%–87.1%) and 82.3% (95% CI: 81.9%–82.7%), respectively. Furthermore, in three-generation households, an allocation of four doses to both parents and two grandchildren was more effective at reducing secondary cases (87.1%, 95% CI: 86.7%–87.4%) than distributing five doses among both grandparents, both parents, and one grandchild (82.5%, 95% CI: 81.9%–83.0%) (Fig. 5A and C). Importantly, these optimal prioritization strategies across all vaccination coverage levels were fully consistent with the results observed in Kunming (Fig. 5B and D).

**Fig. 5:**
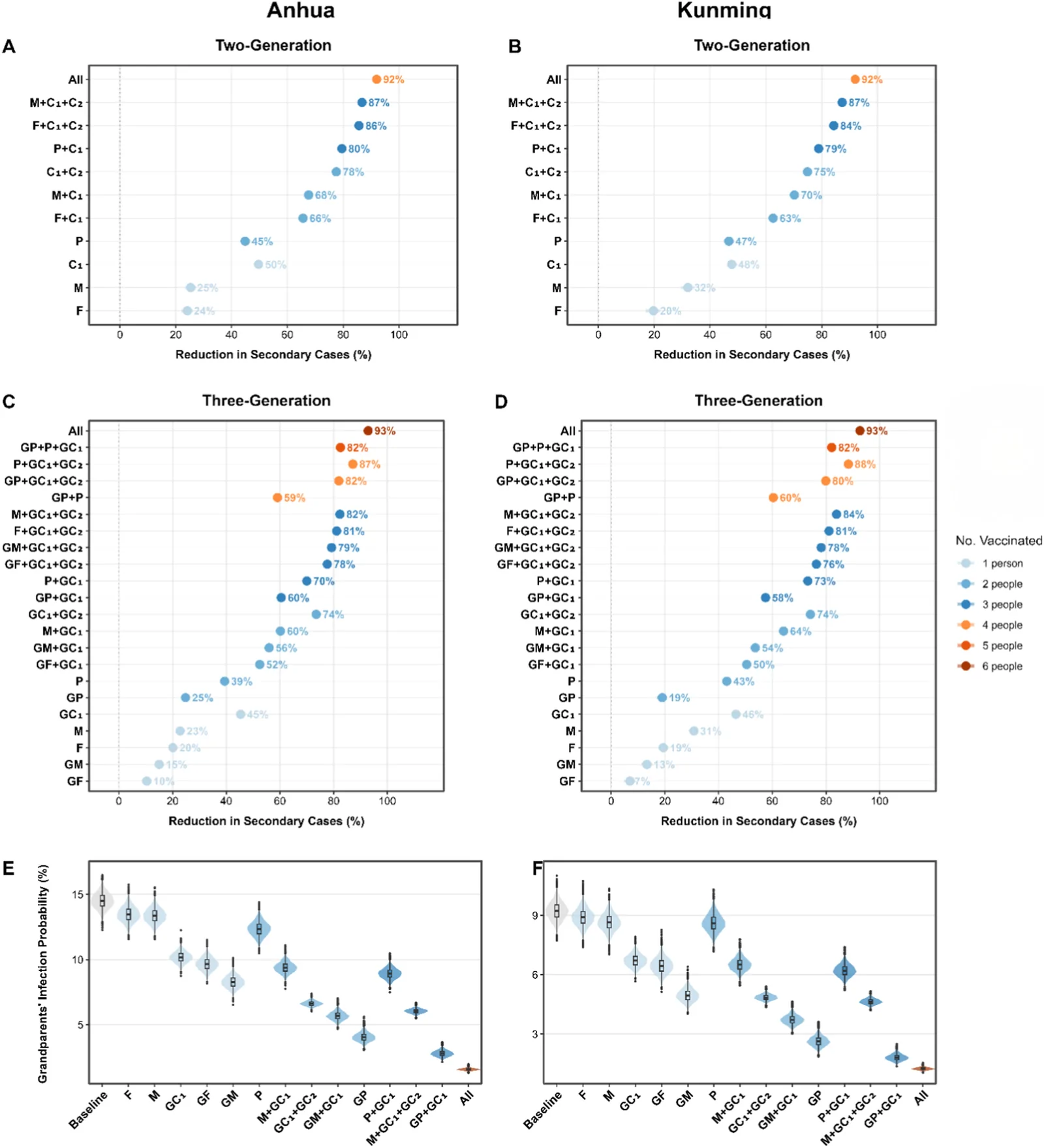
Impact of different vaccination strategies on the reduction of secondary cases and grandparents' infection probability. **(A–D)** Estimated percentage reduction in secondary cases under various vaccination strategies for two-generation (A, Anhua; B, Kunming) and three-generation (C, Anhua; D, Kunming) households. **(E, F)** Grandparents' infection probability under selected strategies in three-generation households in Anhua (E) and Kunming (F). In **(A–D)**, points and horizontal line segments represent the estimated mean reductions and corresponding 95% confidence intervals. Point colors denote the total number of vaccinated individuals per strategy. In **(E)** and **(F)**, violin plots illustrate the probability density of grandparents' infection risk from bootstrapped simulations. Box plots within the violins display the median, 25th and 75th percentiles. Fill colors indicate the number of vaccinated individuals, with gray representing the baseline scenario (no vaccination). Abbreviations: F, father; M, mother; GF, grandfather; GM, grandmother; C_1_/C_2_, children; GC_1_/GC_2_, grandchildren; P, parents; GP, grandparents.

Based on the model results for three-generation households in Anhua County, the baseline probability of infection among grandparents without any household vaccination was 14.52% (95% CI: 13.39%–15.69%). To mitigate this risk under a single-dose constraint, vaccinating the grandmother proved more effective than vaccinating the grandfather, reducing the grandparents' infection probability to 8.28% (95% CI: 7.45%–9.20%). Allocating vaccines to both grandparents further decreased their overall infection probability to 4.05% (95% CI: 3.45%–4.73%). Notably, a combined strategy of vaccinating both grandparents alongside two grandchildren reduced the probability to 1.86% (95% CI: 1.67%–2.06%), which closely approximated the maximum protection achieved by vaccinating the entire household (to 1.58%, 95% CI: 1.41%–1.76%) (Fig. 5E). These strategic rankings and trends observed in Anhua were fully consistent with those in Kunming (Fig. 5F).

### 3.5 Sensitivity analysis

The optimal vaccination strategies remained unchanged across relative susceptibility values of 1.0–3.0 for children and grandchildren (Fig. S17) and alternative father–mother SAR calibration targets of 5%, 8%, 20%, and 30% (Fig. S18). The results of three sensitivity analyses of role-specific external infection risks demonstrated that the strategy of prioritizing children or grandchildren for vaccination remained robust (Figs. S19–S21). The optimal two-dose strategy also generally involved vaccinating children or grandchildren. The only exception occurred when the external infection risks of all household roles were varied simultaneously in Kunming two-generation households. Vaccinating two children remained optimal in 91.74% of the sampled scenarios, whereas the optimal strategy changed to vaccinating the mother and one child in 8.26% of the scenarios, in which the mother's external infection risk ranged from 2.94% to 7.00% and the child's external infection risk ranged from 9.00% to 16.59% (Fig. S21). When only grandparent risks were varied, the three-dose strategy changed at 4.45-fold in Anhua and 7.10-fold in Kunming, while the four-dose strategy changed at 5.55-fold and 9.25-fold, respectively (Fig. S19); The ranking of vaccination strategies remained robust to variations in both vaccine efficacy against susceptibility, vaccine efficacy against infectivity (Figs. S22–S23). The corresponding variations in reduction of secondary cases under these model parameters were detailed in Fig. S24–S27.

## 4 Discussion

By stratifying households into one-, two-, and three-generation structures, this study quantified role-specific household contact patterns. Our data revealed that contact duration between mother-child pairs exceeded that of father-child pairs, consistent with prior studies (Dorélien et al., 2021; Goeyvaerts et al., 2018; Ozella et al., 2018; Rolls et al., 2015). A plausible reason is that mothers shoulder a greater proportion of domestic and childcare responsibilities. Notably, household structure had contrasting impacts on per capita contact and co-presence duration across the two sites. Specifically, in Anhua, these metrics were higher in three-generation households compared to one-generation counterparts. This difference may be attributed to distinct labor pattern and residential environment. A previous study demonstrated that mothers occupied with full-time agricultural fieldwork could readily accommodate their caregiving responsibilities (Chen et al., 2011). In rural areas, family members frequently engage in agricultural activities together and live in more open courtyards, naturally increasing per capita contact and co-presence duration.

Our simulations indicated that mothers as the index cases generated the highest average number of secondary cases. Although model parameter of susceptibility was derived from the literature, extensive evidence shows that children are more susceptible than adults (Cauchemez et al., 2011; Gordon et al., 2018; Cauchemez, 2009; Lau et al., 2015; Tsang et al., 2022; Tsang et al., 2023; Viboud et al., 2004). The mothers' ranking changed only when their relative susceptibilities were equal, which reinforces our finding. Moreover, grandmothers triggered more secondary infections than grandfathers. These findings support the conclusion of an Australian study showing higher intra-household transmission rates for female index cases (McCaw et al., 2012), largely because women are typically at the center of the household contact network. In contrast, grandfathers acting as index cases resulted in the fewest secondary cases. This may explain the observations of COVID-19 household transmission, where the entire household was infected, with the sole exception of the grandfather (Lin et al., 2021). Such a phenomenon is likely due to grandfathers frequently occupying peripheral positions within the household contact network. Our findings offer a new perspective on why household SAR might drop in larger households (e.g., >4 members) (Gordon et al., 2018). Specifically, if the increase in household size is driven by the addition of peripheral members with lower contact frequencies (such as grandfathers), the SAR within the household is effectively diluted. It is worth noting that a Japanese study on influenza identified children as the most likely index cases to infect their parents (Kamigaki et al., 2015), which differs from our observations. This divergence is likely attributable to variations in household composition across the two study populations. Additionally, we emphasized the crowded living environment may amplify the household transmission risk, corroborating evidence that household crowding exacerbates the risk of lower respiratory tract infections in young children (Cardoso et al., 2004). The 0.60 threshold used to define household crowding was derived empirically from the sample median, but sensitivity analyses confirmed that the results were robust.

Our findings provide significant insights into the optimization of household influenza vaccine allocation. Studies consistently show children frequently introduce influenza into the home (Chang et al., 2011; Grijalva et al., 2024; Scott et al., 2019). Extensive evidence demonstrates that vaccinating school-aged children not only mitigates community-wide incidence (Worby et al., 2015) but also significantly reduces household transmission (Gattás et al., 2015), providing crucial indirect protection to vulnerable younger siblings (Tran et al., 2014). Mathematical modeling further supports this, suggesting that a mere 20% vaccination coverage among children can reduce total infections by nearly half (Weycker et al., 2005), solidifying child-targeted vaccination as a highly effective strategy (House et al., 2009). While these studies support our conclusion, our study offers a broader perspective: prioritizing the 'younger generations' (i.e., children and grandchildren) should be based on their family roles, extending beyond traditional age-based vaccination strategies. Furthermore, our simulation results indicated that vaccinating grandparents had little effect on reducing secondary cases, aligning with a prior study which found that influenza vaccination exclusively targeting adults over 60 yields lower systemic economic benefits (Yan et al., 2021). Nevertheless, we also found prioritizing the direct vaccination of grandparents was the most effective approach to reducing their own probability of infection. As previous research highlights, to reduce disease severity and mortality, it is recommended to vaccinate adults over 65 years of age (Zhao et al., 2021). Contact data in Anhua were collected during a period when no local COVID-19 cases were reported and no strict control measures were implemented. Although a previous study indicated that contacts among household members remained relatively stable(Drolet et al., 2022), our results should not be overgeneralized due to the specific timing of the survey.

Several limitations of this study should be acknowledged. First, relying on a single contact pair from the literature for calibration may affect the specific transmission probabilities. In addition, considering grandparents in Anhua County engaging more activities outside the household, their actual external infection risks may exceed dose-specific flipping thresholds in sensitivity analyses, potentially changing the exact optimal vaccination strategy at a given dose level. Moreover, we simplified the household transmission model by not considering between-household transmission. Incorporating between-household transmission in future models would allow a more comprehensive evaluation of vaccination strategies. Importantly, due to differences in the measurement scales for contact duration and the different phases of COVID-19 control policies during the survey periods in Anhua County and Kunming City, the comparability between the two sites is limited, and their differences cannot be attributed solely to urban-rural disparities. Furthermore, recall bias was unavoidable and the cross-sectional study cannot capture seasonal variations in household contact patterns. Given that the exact contact duration in Anhua remains unknown, the estimates of transmission risk may have been affected. Future longitudinal studies are recommended to track changes in contact patterns among household members before and after infection, which could provide more precise parameters for disease modelling.

## 5 Conclusions

In conclusion, our study quantified the heterogeneity in role-specific contact patterns across different household structures in urban and rural areas of China. By developing a within-household influenza transmission model, we identified mothers, when acting as the index cases, generated the highest number of secondary infections. Furthermore, while vaccinating children or grandchildren is the most effective strategy for reducing overall secondary cases, the direct vaccination of grandparents remains the priority for minimizing their own risk of infection.

## CRediT authorship contribution statement

**Tingting Guo:** Writing – review & editing, Writing – original draft, Visualization, Validation, Software, Methodology, Formal analysis, Data curation, Conceptualization. **Bowen Ming:** Writing – review & editing, Visualization, Validation. **Yuxia Liang:** Investigation, Data curation. **Fayu Lu:** Validation. **Wenjing Ma:** Validation, Investigation. **Juanjuan Zhang:** Writing – review & editing, Visualization, Validation, Supervision, Resources, Project administration, Funding acquisition, Conceptualization.

## Informed consent statement

All adult participants provided written informed consent; for minor participants, a family member provided consent on their behalf.

## Institutional Review Board statement

The study was conducted in accordance with the Declaration of Helsinki, and approved by the Institutional Review Board of Fudan University (2020–11–0857, approval date: 5 November 2020; 2023-02-1026, approval date: 3 February 2023).

## Data availability statement

Data are available from the corresponding author upon reasonable request.

## Funding

This study was supported by grants from the 10.13039/501100001809National Natural Science Foundation of China (92369118 and 82304202) and the 10.13039/501100003399Shanghai Municipal Science and Technology Major Project (No. ZD2021CY001).

## Declaration of competing interest

The authors declare that they have no known competing financial interests or personal relationships that could have appeared to influence the work reported in this paper.
