# Supplementary material for: Role-specific household contact patterns in urban and rural China: Implications for influenza household transmission
Source: Infect Dis Model. 2026 Sep 2;12(1):154–69. doi: 10.1016/j.idm.2026.09.001 (PMC13634509; doi:10.1016/j.idm.2026.09.001)
Supplement: Supplementary file 1 [file mmc1.docx]

**Supplementary** **Table 1. Descriptive Statistics with Bootstrap Mean (95% CI)**

|  | **Anhua** | | | | | | **Kunming** | | | | | |
| --- | --- | --- | --- | --- | --- | --- | --- | --- | --- | --- | --- | --- |
| **Variable** | **N** | **Contact Number** | **Total Contact Duration** | **Total**  **Co-presence Duration** | **Per capita contact duration** | **Per capita co-presence duration** | **N** | **Contact Number** | **Total Contact Duration** | **Total**  **Co-presence Duration** | **Per capita contact duration** | **Per capita co-presence duration** |
| **Overall** | **1227 (100.00%)** | **2.58 (2.50, 2.66)** | **5.91 (5.69, 6.15)** | **25.43 (24.43, 26.48)** | **2.27 (2.20, 2.35)** | **10.09 (9.73, 10.45)** | **985 (100.00%)** | **2.26 (2.19, 2.33)** | **7.77 (7.23, 8.33)** | **16.75 (15.85, 17.63)** | **3.54 (3.30, 3.78)** | **7.76 (7.40, 8.11)** |
| **Age group** |  |  |  |  |  |  |  |  |  |  |  |  |
| 0-2 yrs | 107 (8.72%) | 3.01 (2.79, 3.24) | 8.90 (8.20, 9.65) | 39.18 (36.12, 42.33) | 3.01 (2.85, 3.17) | 13.54 (12.71, 14.44) | 50 (5.08%) | 2.70 (2.44, 2.98) | 25.24 (22.34, 27.99) | 39.95 (35.53, 44.47) | 9.91 (8.72, 11.26) | 15.37 (13.91, 16.68) |
| 3-6 yrs | 201 (16.38%) | 3.03 (2.86, 3.21) | 8.32 (7.83, 8.86) | 36.37 (34.20, 38.69) | 2.84 (2.70, 2.97) | 12.82 (12.08, 13.59) | 61 (6.19%) | 2.79 (2.51, 3.07) | 16.79 (14.25, 19.52) | 29.25 (25.31, 33.67) | 6.68 (5.63, 7.77) | 11.18 (9.89, 12.42) |
| 7-19 yrs | 149 (12.14%) | 2.36 (2.13, 2.57) | 4.58 (3.88, 5.27) | 18.20 (15.47, 21.02) | 1.72 (1.49, 1.93) | 6.92 (5.96, 7.80) | 186 (18.88%) | 2.32 (2.17, 2.47) | 5.22 (4.29, 6.10) | 13.46 (11.63, 15.24) | 2.26 (1.87, 2.66) | 5.63 (4.90, 6.33) |
| 20-39 yrs | 322 (26.24%) | 3.02 (2.86, 3.19) | 6.68 (6.18, 7.18) | 27.15 (24.98, 29.27) | 2.19 (2.04, 2.33) | 9.05 (8.37, 9.76) | 183 (18.58%) | 2.32 (2.15, 2.50) | 9.40 (8.09, 10.77) | 17.84 (15.88, 19.96) | 3.99 (3.45, 4.56) | 7.57 (6.84, 8.36) |
| 40-59 yrs | 309 (25.18%) | 2.06 (1.92, 2.21) | 4.39 (4.02, 4.77) | 19.17 (17.52, 20.75) | 2.19 (2.02, 2.36) | 9.60 (8.83, 10.38) | 229 (23.25%) | 2.17 (2.03, 2.34) | 6.62 (5.85, 7.39) | 14.38 (12.98, 15.82) | 3.27 (2.85, 3.68) | 6.94 (6.32, 7.52) |
| 60-75 yrs | 112 (9.13%) | 1.95 (1.71, 2.21) | 3.31 (2.85, 3.78) | 17.03 (14.54, 19.63) | 1.96 (1.70, 2.23) | 10.13 (8.66, 11.63) | 223 (22.64%) | 2.14 (1.96, 2.34) | 4.66 (3.95, 5.32) | 14.20 (12.84, 15.65) | 2.69 (2.23, 3.13) | 8.18 (7.40, 9.06) |
| > 75 yrs | 27 (2.20%) | 1.93 (1.48, 2.44) | 2.44 (1.67, 3.28) | 15.24 (11.70, 18.96) | 1.52 (0.94, 2.15) | 11.24 (7.78, 14.78) | 53 (5.38%) | 1.70 (1.42, 2.04) | 2.36 (1.43, 3.42) | 9.23 (7.22, 11.25) | 1.61 (0.97, 2.33) | 6.54 (4.75, 8.33) |
| **Sex** |  |  |  |  |  |  |  |  |  |  |  |  |
| Male | 503 (40.99%) | 2.65 (2.53, 2.79) | 5.99 (5.64, 6.41) | 25.22 (23.68, 27.01) | 2.21 (2.09, 2.33) | 9.65 (9.09, 10.20) | 468 (47.51%) | 2.28 (2.16, 2.39) | 7.67 (6.88, 8.50) | 16.57 (15.27, 17.88) | 3.59 (3.22, 3.96) | 7.82 (7.27, 8.37) |
| Female | 724 (59.01%) | 2.53 (2.42, 2.63) | 5.85 (5.56, 6.15) | 25.57 (24.28, 26.89) | 2.32 (2.22, 2.41) | 10.39 (9.92, 10.86) | 517 (52.49%) | 2.25 (2.14, 2.35) | 7.87 (7.14, 8.61) | 16.91 (15.77, 18.14) | 3.50 (3.19, 3.83) | 7.71 (7.26, 8.23) |
| **Occupation** |  |  |  |  |  |  |  |  |  |  |  |  |
| Enrolled in pre-kindergarten | 155 (12.63%) | 3.01 (2.82, 3.19) | 8.54 (7.96, 9.16) | 38.54 (36.09, 41.27) | 2.87 (2.70, 3.01) | 13.14 (12.31, 13.90) | 54 (5.48%) | 2.69 (2.43, 2.94) | 24.38 (21.20, 27.43) | 39.25 (34.78, 43.56) | 9.76 (8.43, 11.18) | 15.24 (13.95, 16.66) |
| Enrolled in kindergarten | 135 (11.00%) | 3.09 (2.88, 3.30) | 8.73 (8.11, 9.35) | 36.79 (33.97, 39.48) | 2.95 (2.80, 3.10) | 12.93 (11.97, 13.81) | 37 (3.76%) | 2.62 (2.30, 2.95) | 16.45 (13.66, 19.17) | 27.51 (22.90, 32.25) | 6.80 (5.73, 7.88) | 11.07 (9.56, 12.47) |
| Student | 166 (13.53%) | 2.40 (2.18, 2.61) | 4.82 (4.17, 5.47) | 19.68 (16.84, 22.33) | 1.82 (1.62, 2.02) | 7.64 (6.66, 8.66) | 215 (21.83%) | 2.40 (2.24, 2.54) | 6.28 (5.24, 7.37) | 15.08 (13.19, 17.06) | 2.57 (2.19, 3.01) | 6.06 (5.41, 6.71) |
| School staff | 36 (2.93%) | 2.67 (2.19, 3.17) | 5.61 (4.27, 7.21) | 18.11 (13.33, 23.14) | 2.08 (1.64, 2.48) | 6.61 (4.92, 8.50) | 8 (0.81%) | 1.50 (0.75, 2.12) | 4.61 (1.30, 9.00) | 10.00 (4.31, 16.94) | 2.85 (0.59, 5.62) | 5.49 (2.03, 9.52) |
| Farmer | 334 (27.22%) | 2.10 (1.95, 2.24) | 4.09 (3.75, 4.44) | 19.46 (17.90, 21.24) | 2.06 (1.90, 2.22) | 10.07 (9.25, 10.91) | 18 (1.83%) | 2.33 (1.72, 2.94) | 4.30 (1.70, 7.39) | 13.06 (7.27, 20.17) | 1.51 (0.68, 2.49) | 5.24 (3.07, 7.72) |
| Self-employed | 37 (3.02%) | 2.54 (2.08, 3.08) | 6.79 (5.45, 8.30) | 24.78 (19.34, 30.62) | 2.70 (2.32, 3.11) | 10.30 (8.63, 12.00) | 22 (2.23%) | 2.05 (1.64, 2.45) | 5.66 (3.97, 7.36) | 13.73 (10.83, 16.50) | 2.73 (1.87, 3.69) | 7.32 (5.57, 9.01) |
| Other staff | 34 (2.77%) | 2.59 (2.12, 3.12) | 4.61 (3.52, 5.91) | 17.74 (12.85, 24.07) | 1.84 (1.36, 2.35) | 6.92 (5.16, 9.15) | 168 (17.06%) | 2.15 (1.98, 2.32) | 8.15 (7.03, 9.29) | 15.17 (13.51, 17.05) | 3.94 (3.36, 4.54) | 7.24 (6.50, 8.03) |
| Unemployed | 185 (15.08%) | 2.74 (2.52, 2.97) | 6.53 (5.95, 7.13) | 29.29 (26.62, 32.15) | 2.43 (2.23, 2.62) | 11.25 (10.31, 12.22) | 356 (36.14%) | 2.10 (1.98, 2.24) | 5.25 (4.67, 5.90) | 14.21 (13.06, 15.33) | 2.71 (2.40, 3.05) | 7.71 (7.12, 8.32) |
| Others | 145 (11.82%) | 2.72 (2.48, 2.94) | 5.27 (4.60, 5.92) | 20.02 (17.08, 22.84) | 1.86 (1.66, 2.07) | 7.10 (6.21, 7.98) | 107 (10.86%) | 2.44 (2.18, 2.71) | 8.46 (7.01, 10.03) | 17.71 (15.22, 20.36) | 3.91 (3.24, 4.59) | 7.89 (6.89, 8.94) |
| **Education** |  |  |  |  |  |  |  |  |  |  |  |  |
| None | 577 (47.03%) | 2.68 (2.57, 2.80) | 6.75 (6.38, 7.10) | 29.73 (28.15, 31.23) | 2.51 (2.39, 2.61) | 11.45 (10.94, 11.98) | 303 (30.76%) | 2.49 (2.37, 2.61) | 11.39 (10.02, 12.66) | 22.90 (20.85, 24.94) | 4.72 (4.16, 5.26) | 9.50 (8.81, 10.21) |
| Primary school | 264 (21.52%) | 2.03 (1.86, 2.20) | 3.75 (3.35, 4.14) | 17.70 (15.83, 19.56) | 1.92 (1.74, 2.10) | 9.20 (8.31, 10.16) | 145 (14.72%) | 2.23 (2.05, 2.44) | 4.19 (3.38, 5.07) | 11.81 (10.24, 13.46) | 2.13 (1.71, 2.62) | 6.34 (5.48, 7.24) |
| Middle school | 206 (16.79%) | 2.80 (2.59, 3.02) | 5.78 (5.20, 6.36) | 23.46 (21.05, 25.98) | 2.06 (1.89, 2.24) | 8.55 (7.73, 9.36) | 194 (19.70%) | 2.11 (1.92, 2.29) | 5.30 (4.44, 6.31) | 12.80 (11.31, 14.40) | 2.72 (2.25, 3.24) | 6.65 (5.88, 7.50) |
| High school | 111 (9.05%) | 2.88 (2.65, 3.13) | 6.97 (6.21, 7.72) | 28.45 (25.17, 31.98) | 2.49 (2.25, 2.72) | 10.06 (8.94, 11.18) | 168 (17.06%) | 2.20 (2.01, 2.39) | 7.23 (6.18, 8.44) | 15.98 (14.06, 18.18) | 3.31 (2.83, 3.81) | 7.40 (6.67, 8.17) |
| College or above | 69 (5.62%) | 2.65 (2.26, 3.04) | 5.75 (4.68, 6.88) | 20.07 (15.67, 24.46) | 1.95 (1.65, 2.25) | 6.71 (5.33, 8.08) | 175 (17.77%) | 2.10 (1.93, 2.27) | 7.75 (6.66, 8.90) | 15.31 (13.45, 17.07) | 3.78 (3.24, 4.36) | 7.49 (6.66, 8.26) |
| **Family income** |  |  |  |  |  |  |  |  |  |  |  |  |
| <10k | 57 (4.65%) | 1.72 (1.49, 1.95) | 4.53 (3.57, 5.50) | 21.88 (16.81, 26.73) | 2.55 (2.15, 2.95) | 12.29 (10.31, 14.22) | 19 (1.93%) | 1.63 (1.26, 2.00) | 3.17 (1.35, 5.51) | 10.52 (6.89, 14.77) | 2.23 (0.89, 3.76) | 7.01 (4.37, 9.85) |
| 10-50k | 365 (29.75%) | 2.32 (2.20, 2.46) | 5.43 (5.11, 5.78) | 24.63 (22.99, 26.38) | 2.41 (2.27, 2.54) | 11.12 (10.40, 11.82) | 250 (25.38%) | 2.10 (1.94, 2.25) | 6.13 (5.24, 7.03) | 13.87 (12.41, 15.35) | 3.04 (2.61, 3.47) | 7.04 (6.42, 7.71) |
| 50-100k | 261 (21.27%) | 2.98 (2.79, 3.17) | 6.78 (6.23, 7.32) | 28.16 (25.78, 30.44) | 2.29 (2.13, 2.45) | 9.90 (9.18, 10.69) | 353 (35.84%) | 2.22 (2.10, 2.34) | 8.24 (7.41, 9.17) | 17.12 (15.81, 18.58) | 3.79 (3.41, 4.22) | 8.07 (7.50, 8.72) |
| ≥100k | 190 (15.48%) | 3.13 (2.92, 3.34) | 7.67 (7.10, 8.26) | 31.72 (29.00, 34.73) | 2.47 (2.30, 2.64) | 10.18 (9.39, 11.06) | 280 (28.43%) | 2.46 (2.32, 2.59) | 9.85 (8.64, 11.06) | 20.86 (18.85, 22.82) | 4.19 (3.67, 4.70) | 8.82 (8.09, 9.48) |
| Unknown | 267 (21.76%) | 2.56 (2.38, 2.75) | 5.77 (5.25, 6.32) | 25.10 (23.14, 27.20) | 2.23 (2.06, 2.40) | 10.15 (9.38, 10.96) | 83 (8.43%) | 2.39 (2.10, 2.71) | 4.80 (3.28, 6.54) | 11.44 (8.81, 14.35) | 2.08 (1.44, 2.83) | 5.20 (4.12, 6.31) |
| Unwilling to answer | 5 (0.41%) | 2.60 (1.40, 4.20) | 5.21 (0.40, 10.02) | 17.80 (3.20, 34.60) | 1.42 (0.16, 2.89) | 5.67 (2.60, 9.19) | — |  |  |  |  |  |
| Missing | 82 (6.68%) | 1.83 (1.59, 2.09) | 2.65 (1.98, 3.33) | 9.72 (7.31, 12.32) | 1.19 (0.90, 1.48) | 4.42 (3.40, 5.48) | — |  |  |  |  |  |
| **Underlying disease** |  |  |  |  |  |  |  |  |  |  |  |  |
| No | 917 (74.74%) | 2.72 (2.63, 2.81) | 6.51 (6.23, 6.77) | 27.51 (26.33, 28.72) | 2.40 (2.31, 2.48) | 10.35 (9.93, 10.76) | 659 (66.90%) | 2.34 (2.25, 2.44) | 8.80 (8.14, 9.46) | 17.95 (16.86, 19.17) | 3.79 (3.51, 4.10) | 7.69 (7.27, 8.11) |
| Yes | 269 (21.92%) | 2.17 (2.00, 2.33) | 4.38 (3.91, 4.84) | 20.22 (18.17, 22.18) | 2.03 (1.85, 2.21) | 9.86 (8.95, 10.79) | 326 (33.10%) | 2.10 (1.96, 2.24) | 5.70 (4.99, 6.46) | 14.32 (13.11, 15.49) | 3.03 (2.63, 3.46) | 7.89 (7.27, 8.52) |
| Unknown | 8 (0.65%) | 1.88 (1.38, 2.50) | 4.06 (1.90, 6.71) | 21.71 (6.83, 39.96) | 2.05 (1.14, 2.98) | 10.07 (4.18, 16.72) | — |  |  |  |  |  |
| Unwilling to answer | 33 (2.69%) | 2.00 (1.52, 2.52) | 2.24 (1.41, 3.21) | 11.00 (6.98, 15.55) | 0.95 (0.59, 1.38) | 4.76 (3.35, 6.29) | — |  |  |  |  |  |
| **Day type** |  |  |  |  |  |  |  |  |  |  |  |  |
| Work day | 811 (66.10%) | 2.65 (2.55, 2.75) | 6.03 (5.74, 6.33) | 24.92 (23.65, 26.21) | 2.24 (2.14, 2.33) | 9.50 (9.07, 9.91) | 673 (68.32%) | 2.18 (2.09, 2.27) | 7.24 (6.60, 7.93) | 15.41 (14.41, 16.48) | 3.43 (3.13, 3.75) | 7.44 (7.01, 7.88) |
| Weekend | 416 (33.90%) | 2.44 (2.30, 2.58) | 5.68 (5.30, 6.09) | 26.42 (24.73, 28.29) | 2.35 (2.22, 2.49) | 11.24 (10.59, 11.98) | 312 (31.68%) | 2.43 (2.29, 2.58) | 8.93 (7.91, 9.98) | 19.65 (17.88, 21.52) | 3.78 (3.37, 4.22) | 8.44 (7.76, 9.07) |
| **Recall accuracy** |  |  |  |  |  |  |  |  |  |  |  |  |
| Not well | 16 (1.30%) | 2.25 (1.62, 2.94) | 4.71 (3.04, 6.45) | 21.01 (13.69, 28.44) | 2.45 (1.74, 3.10) | 10.22 (6.86, 13.54) | 3 (0.30%) | 1.33 (1.00, 2.00) | 6.15 (0.20, 11.00) | 13.75 (10.50, 16.00) | 4.32 (0.20, 7.25) | 11.08 (8.00, 14.75) |
| Moderate well | 107 (8.72%) | 2.31 (2.00, 2.60) | 5.16 (4.26, 6.06) | 19.18 (15.67, 22.49) | 1.95 (1.65, 2.22) | 7.72 (6.41, 9.06) | 110 (11.17%) | 2.27 (2.03, 2.51) | 7.39 (5.69, 9.43) | 17.01 (13.71, 20.38) | 3.31 (2.62, 4.06) | 7.45 (6.32, 8.61) |
| Well | 418 (34.07%) | 2.56 (2.42, 2.69) | 5.75 (5.38, 6.12) | 24.64 (22.97, 26.26) | 2.24 (2.11, 2.37) | 9.99 (9.32, 10.62) | 347 (35.23%) | 2.40 (2.27, 2.54) | 7.22 (6.44, 8.03) | 16.71 (15.40, 17.99) | 3.16 (2.81, 3.53) | 7.50 (6.87, 8.07) |
| Very well | 684 (55.75%) | 2.64 (2.53, 2.75) | 6.17 (5.87, 6.50) | 27.04 (25.65, 28.48) | 2.35 (2.26, 2.45) | 10.53 (10.08, 11.03) | 525 (53.30%) | 2.17 (2.06, 2.28) | 8.23 (7.38, 9.04) | 16.74 (15.45, 18.09) | 3.83 (3.47, 4.17) | 7.98 (7.42, 8.48) |
| Unknown | 2 (0.16%) | 2.00 (1.00, 3.00) | 0.14 (0.04, 0.24) | 8.05 (0.10, 16.00) | 0.06 (0.04, 0.08) | 2.72 (0.10, 5.33) | — |  |  |  |  |  |
| **Typical day** |  |  |  |  |  |  |  |  |  |  |  |  |
| No | 108 (8.80%) | 2.69 (2.38, 3.01) | 5.65 (4.81, 6.47) | 24.51 (20.83, 28.58) | 1.97 (1.71, 2.22) | 8.83 (7.57, 10.09) | 123 (12.49%) | 2.46 (2.25, 2.69) | 9.86 (8.12, 11.66) | 21.62 (18.92, 24.76) | 4.09 (3.27, 4.91) | 9.03 (7.87, 10.24) |
| Yes | 1117 (91.04%) | 2.57 (2.49, 2.65) | 5.93 (5.69, 6.19) | 25.50 (24.54, 26.61) | 2.30 (2.22, 2.38) | 10.21 (9.77, 10.62) | 860 (87.31%) | 2.23 (2.15, 2.31) | 7.48 (6.91, 8.02) | 16.06 (15.12, 16.98) | 3.46 (3.20, 3.74) | 7.58 (7.20, 7.98) |
| Unknown | 2 (0.16%) | 3.00 (3.00, 3.00) | 6.12 (0.24, 12.00) | 32.67 (16.00, 49.33) | 2.04 (0.08, 4.00) | 10.89 (5.33, 16.44) | 2 (0.20%) | 1.50 (1.00, 2.00) | 6.25 (0.50, 12.00) | 14.00 (1.00, 27.00) | 3.25 (0.50, 6.00) | 7.25 (1.00, 13.50) |
| **Household Structure** |  |  |  |  |  |  |  |  |  |  |  |  |
| One-Gen | 136 (11.08%) | 0.94 (0.90, 0.99) | 2.76 (2.48, 3.05) | 13.20 (11.89, 14.64) | 2.73 (2.46, 3.02) | 13.06 (11.75, 14.43) | 175 (17.77%) | 0.95 (0.92, 0.99) | 4.54 (3.84, 5.25) | 11.00 (10.03, 11.96) | 4.51 (3.81, 5.21) | 10.96 (9.97, 11.93) |
| Two-Gen | 431 (35.13%) | 2.10 (2.00, 2.20) | 4.81 (4.48, 5.15) | 20.36 (18.79, 21.85) | 2.15 (2.01, 2.27) | 9.39 (8.74, 10.04) | 430 (43.65%) | 1.97 (1.90, 2.04) | 7.34 (6.62, 8.10) | 15.11 (13.94, 16.33) | 3.58 (3.24, 3.95) | 7.36 (6.84, 7.89) |
| Three-Gen | 654 (53.30%) | 3.25 (3.14, 3.37) | 7.33 (6.99, 7.65) | 31.51 (30.05, 32.93) | 2.28 (2.18, 2.37) | 10.00 (9.55, 10.42) | 374 (37.97%) | 3.23 (3.11, 3.35) | 9.91 (8.88, 10.87) | 21.48 (19.88, 23.19) | 3.09 (2.77, 3.41) | 6.79 (6.30, 7.28) |
| Others | 6 (0.49%) | 1.00 (0.33, 1.67) | 1.55 (0.01, 4.21) | 4.17 (0.50, 10.67) | 0.78 (0.01, 2.11) | 2.33 (0.33, 5.50) | 6 (0.61%) | 1.00 (0.17, 1.83) | 0.61 (0.03, 1.58) | 7.00 (1.00, 14.00) | 0.31 (0.03, 0.78) | 3.33 (1.00, 6.00) |
| **Family role** |  |  |  |  |  |  |  |  |  |  |  |  |
| Father | 140 (11.41%) | 2.44 (2.17, 2.69) | 5.85 (5.21, 6.51) | 23.95 (21.24, 27.04) | 2.53 (2.30, 2.76) | 11.04 (9.90, 12.20) | 201 (20.41%) | 1.86 (1.70, 2.02) | 7.33 (6.39, 8.24) | 15.45 (13.97, 16.98) | 4.25 (3.72, 4.82) | 9.26 (8.42, 10.11) |
| Mother | 370 (30.15%) | 2.35 (2.19, 2.50) | 5.56 (5.15, 5.96) | 24.57 (22.75, 26.36) | 2.43 (2.29, 2.56) | 10.97 (10.30, 11.64) | 257 (26.09%) | 1.86 (1.71, 2.00) | 7.49 (6.57, 8.46) | 15.48 (13.98, 16.90) | 3.78 (3.33, 4.24) | 8.40 (7.71, 9.08) |
| Child | 244 (19.89%) | 2.10 (1.95, 2.25) | 4.18 (3.71, 4.65) | 17.85 (15.59, 19.98) | 1.80 (1.61, 1.98) | 7.86 (6.99, 8.73) | 242 (24.57%) | 1.97 (1.86, 2.05) | 7.23 (6.10, 8.32) | 15.08 (13.32, 16.81) | 3.61 (3.06, 4.17) | 7.35 (6.56, 8.12) |
| Grandfather | 41 (3.34%) | 3.17 (2.73, 3.68) | 4.98 (4.03, 5.95) | 22.31 (18.31, 26.36) | 1.76 (1.38, 2.13) | 7.45 (5.88, 8.94) | 63 (6.40%) | 3.44 (3.11, 3.76) | 5.06 (3.78, 6.55) | 17.65 (14.53, 21.04) | 1.44 (1.09, 1.88) | 5.61 (4.62, 6.57) |
| Grandmother | 83 (6.76%) | 3.02 (2.73, 3.31) | 6.11 (5.27, 7.01) | 28.55 (24.57, 32.56) | 2.10 (1.80, 2.42) | 10.31 (8.74, 11.91) | 90 (9.14%) | 3.14 (2.86, 3.42) | 5.71 (4.44, 7.04) | 15.21 (12.40, 18.10) | 1.73 (1.33, 2.13) | 4.76 (3.94, 5.65) |
| Grandchild | 334 (27.22%) | 3.06 (2.91, 3.21) | 7.73 (7.30, 8.21) | 32.71 (30.85, 34.76) | 2.50 (2.38, 2.62) | 10.88 (10.24, 11.49) | 120 (12.18%) | 3.13 (2.93, 3.32) | 13.86 (11.75, 16.21) | 26.02 (22.80, 29.69) | 4.48 (3.76, 5.26) | 8.34 (7.38, 9.35) |
| Others | 15 (1.22%) | 2.67 (1.73, 3.67) | 4.16 (2.12, 6.25) | 12.75 (7.51, 18.51) | 1.18 (0.63, 1.84) | 3.96 (2.37, 5.67) | 12 (1.22%) | 2.00 (1.17, 2.83) | 0.99 (0.42, 1.71) | 13.57 (7.50, 20.42) | 0.44 (0.15, 0.82) | 5.26 (2.96, 7.96) |
| **Household size** |  |  |  |  |  |  |  |  |  |  |  |  |
| 2 | 216 (17.60%) | 0.93 (0.89, 0.96) | 2.53 (2.32, 2.76) | 12.13 (11.02, 13.23) | 2.53 (2.32, 2.76) | 12.13 (11.02, 13.23) | 230 (23.35%) | 0.95 (0.92, 0.97) | 3.97 (3.40, 4.55) | 9.41 (8.57, 10.34) | 3.97 (3.40, 4.55) | 9.41 (8.57, 10.34) |
| 3 | 316 (25.75%) | 1.81 (1.76, 1.86) | 4.33 (4.01, 4.65) | 19.32 (17.72, 20.78) | 2.23 (2.07, 2.39) | 10.10 (9.27, 10.84) | 286 (29.04%) | 1.79 (1.73, 1.85) | 6.28 (5.45, 7.10) | 13.21 (11.96, 14.43) | 3.41 (2.97, 3.85) | 7.29 (6.61, 7.95) |
| 4 | 324 (26.41%) | 2.67 (2.59, 2.74) | 6.51 (6.11, 6.96) | 29.09 (27.22, 31.07) | 2.33 (2.19, 2.47) | 10.52 (9.86, 11.22) | 237 (24.06%) | 2.54 (2.45, 2.64) | 9.77 (8.58, 10.91) | 19.40 (17.62, 21.12) | 3.77 (3.31, 4.24) | 7.56 (6.87, 8.17) |
| 5 | 168 (13.69%) | 3.51 (3.36, 3.64) | 7.50 (6.90, 8.09) | 31.85 (29.34, 34.56) | 2.11 (1.94, 2.28) | 8.92 (8.17, 9.69) | 128 (12.99%) | 3.43 (3.28, 3.56) | 11.44 (9.73, 13.30) | 24.87 (22.08, 27.82) | 3.36 (2.81, 3.94) | 7.30 (6.48, 8.15) |
| >5 | 203 (16.54%) | 4.61 (4.40, 4.81) | 9.68 (9.01, 10.32) | 37.92 (35.10, 40.67) | 2.11 (1.96, 2.24) | 8.18 (7.57, 8.73) | 104 (10.56%) | 4.36 (4.14, 4.55) | 11.22 (9.24, 13.20) | 26.69 (23.17, 30.13) | 2.65 (2.15, 3.15) | 6.43 (5.55, 7.29) |
| **Bedrooms in use** |  |  |  |  |  |  |  |  |  |  |  |  |
| 1 | 322 (26.24%) | 1.60 (1.51, 1.70) | 5.33 (4.96, 5.71) | 25.83 (24.10, 27.56) | 3.17 (3.01, 3.32) | 15.50 (14.75, 16.26) | — |  |  |  |  |  |
| 2 | 511 (41.65%) | 2.49 (2.38, 2.59) | 5.72 (5.35, 6.10) | 24.81 (23.19, 26.39) | 2.08 (1.97, 2.18) | 8.99 (8.45, 9.50) | — |  |  |  |  |  |
| 3 | 283 (23.06%) | 3.27 (3.09, 3.44) | 6.46 (5.90, 6.99) | 25.45 (23.12, 27.65) | 1.83 (1.69, 1.97) | 7.22 (6.66, 7.75) | — |  |  |  |  |  |
| 4 | 71 (5.79%) | 4.01 (3.65, 4.41) | 7.57 (6.30, 8.83) | 27.50 (23.59, 31.80) | 1.87 (1.58, 2.15) | 6.78 (5.84, 7.83) | — |  |  |  |  |  |
| >4 | 29 (2.36%) | 4.93 (4.21, 5.66) | 7.09 (5.09, 9.09) | 29.30 (23.35, 36.30) | 1.44 (1.09, 1.81) | 6.38 (4.90, 8.19) | — |  |  |  |  |  |
| Unknown | 11 (0.90%) | 2.36 (1.64, 3.27) | 3.33 (1.24, 5.50) | 18.43 (6.45, 30.79) | 1.39 (0.48, 2.44) | 7.62 (2.04, 13.66) | — |  |  |  |  |  |
| **Weather** |  |  |  |  |  |  |  |  |  |  |  |  |
| Sunny | 760 (61.94%) | 2.60 (2.50, 2.70) | 6.05 (5.74, 6.35) | 25.28 (23.97, 26.61) | 2.33 (2.24, 2.42) | 9.95 (9.51, 10.41) | — |  |  |  |  |  |
| Cloudy | 141 (11.49%) | 2.77 (2.53, 3.02) | 6.71 (6.06, 7.44) | 28.51 (25.96, 31.43) | 2.47 (2.27, 2.67) | 11.03 (10.02, 12.05) | — |  |  |  |  |  |
| Rainy | 271 (22.09%) | 2.43 (2.28, 2.60) | 5.22 (4.79, 5.71) | 24.45 (22.52, 26.66) | 2.09 (1.94, 2.25) | 10.13 (9.32, 10.93) | — |  |  |  |  |  |
| Varied | 50 (4.07%) | 2.52 (2.04, 3.06) | 5.32 (4.08, 6.60) | 24.52 (18.69, 30.28) | 1.93 (1.54, 2.31) | 9.75 (7.87, 11.94) | — |  |  |  |  |  |
| Unknown | 5 (0.41%) | 3.20 (2.20, 4.20) | 5.28 (1.71, 9.16) | 22.60 (9.60, 37.00) | 1.50 (0.66, 2.26) | 6.27 (3.47, 8.60) | — |  |  |  |  |  |

**Supplementary** **Table 2. Descriptive Statistics with Median (Q1, Q3)**

|  | **Anhua** | | | | | | **Kunming** | | | | | |
| --- | --- | --- | --- | --- | --- | --- | --- | --- | --- | --- | --- | --- |
| **Variable** | **N** | **Contact Number** | **Total Contact Duration** | **Total**  **Co-presence Duration** | **Per capita contact duration** | **Per capita co-presence duration** | **N** | **Contact Number** | **Total Contact Duration** | **Total**  **Co-presence Duration** | **Per capita contact duration** | **Per capita co-presence duration** |
| **Overall** | **1227 (100.00%)** | **2.00 (2.00, 3.00)** | **4.86 (2.82, 8.39)** | **24.00 (11.00, 38.00)** | **2.37 (1.26, 3.50)** | **9.33 (5.00, 14.00)** | **985 (100.00%)** | **2.00 (1.00, 3.00)** | **4.64 (1.00, 11.08)** | **14.00 (6.00, 23.50)** | **2.00 (0.50, 5.36)** | **7.00 (3.00, 11.60)** |
| **Age group** |  |  |  |  |  |  |  |  |  |  |  |  |
| 0-2 yrs | 107 (8.72%) | 3.00 (2.00, 4.00) | 8.62 (6.50, 12.00) | 38.00 (27.33, 48.00) | 3.00 (2.37, 4.00) | 13.00 (10.50, 16.80) | 50 (5.08%) | 3.00 (2.00, 3.00) | 25.00 (17.25, 32.85) | 36.84 (31.50, 47.38) | 9.50 (6.87, 12.50) | 16.29 (12.00, 17.94) |
| 3-6 yrs | 201 (16.38%) | 3.00 (2.00, 4.00) | 8.00 (6.50, 10.58) | 34.00 (24.00, 48.00) | 2.87 (2.22, 4.00) | 12.00 (9.00, 16.00) | 61 (6.19%) | 3.00 (2.00, 4.00) | 15.00 (8.67, 23.67) | 26.00 (18.00, 34.34) | 5.92 (3.17, 9.00) | 10.33 (7.62, 15.00) |
| 7-19 yrs | 149 (12.14%) | 2.00 (1.00, 3.00) | 4.04 (0.62, 8.00) | 13.00 (3.00, 30.00) | 1.74 (0.23, 2.71) | 6.00 (2.00, 10.00) | 186 (18.88%) | 2.00 (2.00, 3.00) | 2.42 (0.75, 6.99) | 10.00 (2.44, 19.75) | 1.00 (0.50, 3.09) | 4.64 (1.07, 8.86) |
| 20-39 yrs | 322 (26.24%) | 3.00 (2.00, 4.00) | 6.50 (4.00, 10.00) | 24.00 (12.08, 42.00) | 2.23 (1.33, 3.23) | 8.00 (4.29, 12.73) | 183 (18.58%) | 2.00 (1.00, 3.00) | 8.00 (1.54, 15.12) | 15.00 (8.00, 25.00) | 3.17 (0.69, 6.00) | 6.75 (3.83, 11.00) |
| 40-59 yrs | 309 (25.18%) | 2.00 (1.00, 3.00) | 4.00 (1.85, 6.54) | 16.00 (8.00, 27.00) | 2.22 (0.62, 4.00) | 8.00 (4.00, 14.00) | 229 (23.25%) | 2.00 (1.00, 3.00) | 6.25 (1.04, 9.84) | 13.00 (8.00, 19.00) | 2.19 (0.51, 4.75) | 6.19 (3.28, 9.50) |
| 60-75 yrs | 112 (9.13%) | 2.00 (1.00, 3.00) | 4.00 (0.87, 4.12) | 13.75 (6.75, 24.00) | 2.01 (0.46, 4.00) | 7.58 (3.00, 14.25) | 223 (22.64%) | 2.00 (1.00, 3.00) | 2.00 (0.51, 8.33) | 12.00 (6.00, 20.00) | 1.00 (0.33, 3.75) | 7.25 (3.00, 12.00) |
| > 75 yrs | 27 (2.20%) | 1.00 (1.00, 3.00) | 2.54 (0.22, 4.00) | 14.00 (8.00, 24.00) | 1.05 (0.10, 2.87) | 8.00 (2.58, 22.00) | 53 (5.38%) | 1.00 (1.00, 2.00) | 0.83 (0.33, 3.00) | 8.00 (1.32, 15.00) | 0.46 (0.17, 1.62) | 4.00 (1.00, 12.00) |
| **Sex** |  |  |  |  |  |  |  |  |  |  |  |  |
| Male | 503 (40.99%) | 3.00 (2.00, 3.00) | 5.00 (2.50, 8.62) | 22.50 (10.17, 37.00) | 2.35 (0.94, 3.42) | 8.60 (4.25, 13.58) | 468 (47.51%) | 2.00 (1.00, 3.00) | 4.54 (0.95, 11.00) | 14.00 (6.00, 22.12) | 2.00 (0.50, 5.44) | 6.58 (3.00, 12.00) |
| Female | 724 (59.01%) | 2.00 (1.00, 3.00) | 4.78 (3.12, 8.17) | 24.00 (11.00, 38.00) | 2.37 (1.35, 3.52) | 10.00 (5.40, 14.67) | 517 (52.49%) | 2.00 (1.00, 3.00) | 5.00 (1.00, 11.75) | 14.00 (6.00, 24.00) | 2.00 (0.50, 5.33) | 7.06 (3.00, 11.33) |
| **Occupation** |  |  |  |  |  |  |  |  |  |  |  |  |
| Enrolled in pre-kindergarten | 155 (12.63%) | 3.00 (2.00, 4.00) | 8.16 (5.23, 11.57) | 36.00 (26.17, 48.00) | 2.87 (2.31, 3.80) | 12.67 (10.33, 16.55) | 54 (5.48%) | 3.00 (2.00, 3.00) | 24.75 (17.00, 32.31) | 36.34 (29.12, 47.38) | 9.00 (6.58, 12.50) | 16.29 (11.32, 18.64) |
| Enrolled in kindergarten | 135 (11.00%) | 3.00 (2.00, 4.00) | 8.04 (6.66, 11.31) | 33.00 (24.00, 48.00) | 3.00 (2.34, 4.00) | 12.00 (8.55, 16.00) | 37 (3.76%) | 2.00 (2.00, 3.00) | 15.00 (11.62, 23.50) | 24.25 (17.50, 34.00) | 6.50 (4.85, 9.00) | 10.00 (7.88, 15.00) |
| Student | 166 (13.53%) | 2.00 (2.00, 3.00) | 4.08 (0.72, 8.00) | 16.00 (5.00, 31.00) | 2.02 (0.39, 2.83) | 6.58 (2.50, 11.88) | 215 (21.83%) | 2.00 (2.00, 3.00) | 2.67 (0.92, 9.59) | 12.00 (3.00, 22.12) | 1.07 (0.50, 3.29) | 5.00 (1.50, 9.75) |
| School staff | 36 (2.93%) | 2.50 (1.75, 4.00) | 4.76 (2.41, 7.04) | 12.00 (5.50, 28.00) | 2.08 (1.05, 3.25) | 6.00 (2.25, 8.54) | 8 (0.81%) | 2.00 (0.75, 2.00) | 3.00 (0.38, 5.78) | 7.25 (3.75, 15.12) | 1.17 (0.19, 3.64) | 3.62 (1.88, 8.13) |
| Farmer | 334 (27.22%) | 2.00 (1.00, 3.00) | 4.00 (1.23, 5.23) | 16.50 (8.00, 25.96) | 2.02 (0.62, 4.00) | 8.29 (3.21, 15.00) | 18 (1.83%) | 2.00 (1.00, 3.00) | 0.90 (0.17, 6.82) | 13.00 (1.50, 18.16) | 0.40 (0.17, 2.86) | 3.40 (1.07, 8.00) |
| Self-employed | 37 (3.02%) | 2.00 (1.00, 3.00) | 5.85 (4.00, 10.47) | 21.00 (12.00, 30.00) | 2.84 (1.70, 4.00) | 9.00 (6.75, 15.00) | 22 (2.23%) | 2.00 (1.00, 3.00) | 6.33 (1.20, 8.85) | 13.00 (9.88, 16.56) | 2.72 (0.79, 3.60) | 6.50 (4.12, 8.85) |
| Other staff | 34 (2.77%) | 2.00 (1.25, 3.00) | 4.28 (1.23, 7.88) | 15.00 (6.50, 20.75) | 1.80 (0.62, 2.85) | 6.25 (3.52, 7.31) | 168 (17.06%) | 2.00 (1.00, 3.00) | 8.09 (1.50, 12.00) | 12.97 (7.50, 20.00) | 3.17 (0.58, 5.50) | 6.62 (3.72, 10.00) |
| Unemployed | 185 (15.08%) | 2.00 (2.00, 4.00) | 6.00 (4.00, 9.39) | 25.00 (15.00, 44.00) | 2.46 (1.56, 4.00) | 10.40 (7.00, 16.00) | 356 (36.14%) | 2.00 (1.00, 3.00) | 2.00 (0.57, 8.88) | 12.09 (5.38, 20.00) | 1.12 (0.41, 4.00) | 6.95 (3.00, 11.60) |
| Others | 145 (11.82%) | 3.00 (2.00, 4.00) | 4.50 (1.23, 8.04) | 16.00 (7.00, 28.00) | 2.00 (0.62, 2.66) | 6.33 (3.00, 10.00) | 107 (10.86%) | 2.00 (1.00, 3.00) | 8.34 (1.50, 10.61) | 15.00 (9.38, 22.25) | 3.00 (0.51, 5.77) | 7.25 (3.98, 11.75) |
| **Education** |  |  |  |  |  |  |  |  |  |  |  |  |
| None | 577 (47.03%) | 3.00 (2.00, 3.00) | 6.70 (4.00, 9.00) | 28.00 (16.00, 42.00) | 2.65 (1.74, 4.00) | 11.00 (7.00, 15.67) | 303 (30.76%) | 2.00 (2.00, 3.00) | 8.45 (1.96, 18.00) | 19.00 (10.25, 33.88) | 3.17 (0.75, 7.62) | 9.00 (4.88, 14.00) |
| Primary school | 264 (21.52%) | 2.00 (1.00, 3.00) | 4.00 (0.62, 4.78) | 14.25 (4.00, 25.00) | 1.98 (0.27, 4.00) | 8.00 (2.50, 14.00) | 145 (14.72%) | 2.00 (1.00, 3.00) | 1.66 (0.63, 6.96) | 10.21 (3.84, 16.00) | 0.88 (0.35, 3.00) | 4.94 (2.00, 9.00) |
| Middle school | 206 (16.79%) | 2.50 (2.00, 4.00) | 4.86 (2.52, 8.45) | 20.29 (9.62, 33.00) | 2.06 (0.88, 3.00) | 7.27 (3.50, 12.00) | 194 (19.70%) | 2.00 (1.00, 3.00) | 2.00 (0.57, 9.06) | 11.75 (3.00, 19.00) | 1.08 (0.33, 4.08) | 5.58 (2.00, 9.50) |
| High school | 111 (9.05%) | 3.00 (2.00, 4.00) | 6.82 (4.00, 10.50) | 26.00 (13.00, 43.00) | 2.46 (1.65, 4.00) | 9.00 (6.00, 14.00) | 168 (17.06%) | 2.00 (1.00, 3.00) | 5.01 (1.00, 10.51) | 13.00 (7.21, 21.50) | 2.50 (0.52, 5.02) | 6.97 (3.56, 10.87) |
| College or above | 69 (5.62%) | 3.00 (2.00, 4.00) | 4.86 (2.00, 8.47) | 17.00 (4.00, 26.00) | 2.00 (0.97, 2.78) | 5.33 (2.50, 10.80) | 175 (17.77%) | 2.00 (1.00, 3.00) | 6.00 (1.50, 11.04) | 13.58 (6.00, 20.00) | 2.95 (0.66, 5.50) | 6.83 (3.00, 10.92) |
| **Family income** |  |  |  |  |  |  |  |  |  |  |  |  |
| <10k | 57 (4.65%) | 1.00 (1.00, 2.00) | 4.00 (2.50, 4.78) | 18.00 (10.00, 25.00) | 2.50 (1.59, 4.00) | 12.00 (6.00, 20.00) | 19 (1.93%) | 2.00 (1.00, 2.00) | 0.86 (0.62, 1.79) | 8.00 (4.25, 14.75) | 0.61 (0.31, 1.71) | 7.25 (2.25, 9.25) |
| 10-50k | 365 (29.75%) | 2.00 (1.00, 3.00) | 4.62 (4.00, 8.00) | 24.00 (12.50, 36.00) | 2.50 (1.55, 4.00) | 10.33 (6.46, 15.67) | 250 (25.38%) | 2.00 (1.00, 3.00) | 3.98 (0.58, 9.50) | 12.00 (4.00, 19.00) | 1.74 (0.45, 4.62) | 6.00 (2.75, 10.00) |
| 50-100k | 261 (21.27%) | 3.00 (2.00, 4.00) | 6.50 (4.00, 9.62) | 24.00 (14.00, 39.00) | 2.37 (1.37, 3.25) | 8.75 (5.67, 13.50) | 353 (35.84%) | 2.00 (1.00, 3.00) | 6.00 (1.33, 11.67) | 14.75 (8.00, 23.50) | 2.89 (0.67, 5.50) | 6.50 (3.75, 11.88) |
| ≥100k | 190 (15.48%) | 3.00 (2.00, 4.00) | 8.00 (4.39, 10.65) | 30.00 (18.00, 45.00) | 2.55 (1.74, 3.44) | 9.63 (5.85, 14.00) | 280 (28.43%) | 2.00 (1.00, 3.00) | 6.42 (1.29, 15.00) | 17.12 (8.40, 30.62) | 2.88 (0.58, 6.49) | 8.29 (4.00, 12.41) |
| Unknown | 267 (21.76%) | 2.00 (1.00, 4.00) | 4.62 (2.62, 8.62) | 24.00 (10.00, 38.00) | 2.31 (1.02, 3.50) | 10.00 (4.50, 13.75) | 83 (8.43%) | 2.00 (1.00, 3.00) | 1.50 (0.51, 6.98) | 9.00 (3.00, 15.50) | 0.67 (0.26, 2.32) | 3.50 (1.00, 7.75) |
| Unwilling to answer | 5 (0.41%) | 3.00 (1.00, 3.00) | 1.85 (0.04, 12.00) | 4.00 (3.00, 34.00) | 0.62 (0.04, 2.42) | 4.00 (3.00, 9.00) | — |  |  |  |  |  |
| Missing | 82 (6.68%) | 2.00 (1.00, 2.75) | 1.20 (0.13, 4.62) | 5.10 (0.62, 13.00) | 0.61 (0.05, 2.31) | 2.83 (0.30, 6.33) | — |  |  |  |  |  |
| **Underlying disease** |  |  |  |  |  |  |  |  |  |  |  |  |
| No | 917 (74.74%) | 3.00 (2.00, 4.00) | 6.50 (4.00, 9.00) | 25.00 (13.00, 40.00) | 2.50 (1.55, 3.50) | 10.00 (6.00, 14.00) | 659 (66.90%) | 2.00 (1.00, 3.00) | 5.91 (1.17, 13.00) | 14.62 (6.00, 26.00) | 2.50 (0.54, 5.74) | 6.89 (3.00, 11.53) |
| Yes | 269 (21.92%) | 2.00 (1.00, 3.00) | 4.00 (1.13, 6.66) | 17.50 (7.00, 28.00) | 2.02 (0.62, 4.00) | 8.00 (3.00, 16.00) | 326 (33.10%) | 2.00 (1.00, 3.00) | 2.50 (0.67, 9.50) | 13.00 (6.12, 19.38) | 1.35 (0.47, 4.53) | 7.00 (3.25, 11.60) |
| Unknown | 8 (0.65%) | 2.00 (1.00, 2.25) | 3.25 (1.22, 5.76) | 12.50 (2.17, 32.00) | 2.50 (0.61, 2.89) | 6.25 (2.12, 19.33) | — |  |  |  |  |  |
| Unwilling to answer | 33 (2.69%) | 2.00 (1.00, 3.00) | 0.62 (0.16, 4.00) | 8.00 (2.00, 13.00) | 0.62 (0.08, 1.36) | 4.00 (2.00, 6.00) | — |  |  |  |  |  |
| **Day type** |  |  |  |  |  |  |  |  |  |  |  |  |
| Work day | 811 (66.10%) | 2.00 (2.00, 4.00) | 5.23 (2.78, 8.62) | 23.00 (11.00, 37.00) | 2.31 (1.16, 3.42) | 9.00 (4.37, 13.58) | 673 (68.32%) | 2.00 (1.00, 3.00) | 4.00 (0.83, 10.50) | 13.00 (5.50, 21.00) | 2.00 (0.50, 5.25) | 6.33 (3.00, 11.12) |
| Weekend | 416 (33.90%) | 2.00 (1.00, 3.00) | 4.40 (3.08, 8.16) | 24.00 (11.75, 39.75) | 2.50 (1.36, 4.00) | 10.12 (6.31, 16.00) | 312 (31.68%) | 2.00 (1.00, 3.00) | 6.21 (1.07, 13.37) | 16.00 (7.88, 28.00) | 2.50 (0.60, 5.76) | 7.50 (3.27, 12.53) |
| **Recall accuracy** |  |  |  |  |  |  |  |  |  |  |  |  |
| Not well | 16 (1.30%) | 2.00 (1.00, 3.00) | 4.00 (2.21, 6.88) | 19.00 (10.25, 31.00) | 2.77 (2.02, 3.31) | 10.75 (4.45, 15.00) | 3 (0.30%) | 1.00 (1.00, 1.50) | 7.25 (3.73, 9.12) | 14.75 (12.62, 15.38) | 5.50 (2.85, 6.38) | 10.50 (9.25, 12.62) |
| Moderate well | 107 (8.72%) | 2.00 (1.00, 3.50) | 4.00 (0.74, 8.00) | 14.00 (2.50, 32.25) | 1.93 (0.55, 3.42) | 6.00 (2.00, 12.50) | 110 (11.17%) | 2.00 (1.00, 3.00) | 4.12 (0.67, 10.04) | 12.00 (5.12, 24.62) | 1.62 (0.50, 4.51) | 6.13 (2.58, 11.60) |
| Well | 418 (34.07%) | 2.00 (2.00, 3.00) | 5.18 (3.01, 8.16) | 24.00 (11.00, 35.75) | 2.31 (1.29, 3.46) | 9.22 (5.00, 14.00) | 347 (35.23%) | 2.00 (1.00, 3.00) | 4.00 (1.00, 10.61) | 14.77 (6.00, 24.38) | 1.75 (0.50, 4.92) | 6.83 (3.00, 11.00) |
| Very well | 684 (55.75%) | 2.00 (2.00, 3.00) | 5.00 (4.00, 8.69) | 24.00 (13.00, 40.00) | 2.37 (1.44, 4.00) | 10.00 (5.84, 15.00) | 525 (53.30%) | 2.00 (1.00, 3.00) | 5.00 (1.00, 12.29) | 14.00 (6.00, 22.00) | 2.32 (0.50, 5.67) | 7.00 (3.18, 12.00) |
| Unknown | 2 (0.16%) | 2.00 (1.50, 2.50) | 0.14 (0.09, 0.19) | 8.05 (4.08, 12.03) | 0.06 (0.05, 0.07) | 2.72 (1.41, 4.02) | — |  |  |  |  |  |
| **Typical day** |  |  |  |  |  |  |  |  |  |  |  |  |
| No | 108 (8.80%) | 3.00 (1.00, 4.00) | 4.78 (1.23, 8.62) | 21.00 (8.00, 37.25) | 2.21 (0.62, 2.88) | 7.40 (3.85, 12.56) | 123 (12.49%) | 2.00 (2.00, 3.00) | 6.58 (1.71, 15.12) | 18.00 (9.09, 30.75) | 2.95 (0.69, 5.92) | 7.72 (3.75, 14.50) |
| Yes | 1117 (91.04%) | 2.00 (2.00, 3.00) | 5.00 (3.12, 8.32) | 24.00 (11.00, 38.00) | 2.40 (1.33, 3.50) | 9.75 (5.00, 14.25) | 860 (87.31%) | 2.00 (1.00, 3.00) | 4.50 (0.92, 10.50) | 13.54 (6.00, 22.00) | 2.00 (0.50, 5.25) | 6.79 (3.00, 11.27) |
| Unknown | 2 (0.16%) | 3.00 (3.00, 3.00) | 6.12 (3.18, 9.06) | 32.67 (24.33, 41.00) | 2.04 (1.06, 3.02) | 10.89 (8.11, 13.67) | 2 (0.20%) | 1.50 (1.25, 1.75) | 6.25 (3.38, 9.12) | 14.00 (7.50, 20.50) | 3.25 (1.88, 4.62) | 7.25 (4.12, 10.38) |
| **Household Structure** |  |  |  |  |  |  |  |  |  |  |  |  |
| One-Gen | 136 (11.08%) | 1.00 (1.00, 1.00) | 4.00 (0.62, 4.00) | 12.00 (8.00, 21.00) | 4.00 (0.62, 4.00) | 12.00 (8.00, 21.00) | 175 (17.77%) | 1.00 (1.00, 1.00) | 2.00 (0.50, 9.00) | 11.00 (5.50, 16.00) | 2.00 (0.50, 9.00) | 11.00 (5.50, 15.62) |
| Two-Gen | 431 (35.13%) | 2.00 (1.00, 3.00) | 4.08 (1.23, 8.00) | 16.00 (5.75, 30.00) | 2.22 (0.62, 4.00) | 8.00 (3.00, 15.00) | 430 (43.65%) | 2.00 (2.00, 2.00) | 4.00 (0.90, 10.63) | 13.00 (4.00, 21.00) | 2.00 (0.50, 5.31) | 6.42 (2.77, 11.00) |
| Three-Gen | 654 (53.30%) | 3.00 (2.00, 4.00) | 8.00 (4.17, 10.49) | 30.00 (19.00, 44.00) | 2.31 (1.44, 3.15) | 9.67 (6.00, 13.00) | 374 (37.97%) | 3.00 (2.00, 4.00) | 6.64 (1.67, 15.44) | 18.00 (9.51, 30.19) | 2.07 (0.54, 4.60) | 6.00 (3.00, 9.65) |
| Others | 6 (0.49%) | 1.00 (0.25, 1.75) | 0.04 (0.01, 0.94) | 1.50 (0.25, 2.00) | 0.04 (0.01, 0.47) | 1.00 (0.25, 1.75) | 6 (0.61%) | 0.50 (0.00, 1.75) | 0.08 (0.00, 0.42) | 3.00 (0.00, 10.50) | 0.08 (0.00, 0.17) | 3.00 (0.00, 6.00) |
| **Family role** |  |  |  |  |  |  |  |  |  |  |  |  |
| Father | 140 (11.41%) | 2.00 (1.00, 3.00) | 4.34 (4.00, 8.11) | 21.00 (12.00, 30.38) | 2.50 (1.56, 4.00) | 9.54 (6.25, 16.00) | 201 (20.41%) | 2.00 (1.00, 3.00) | 8.00 (1.00, 10.33) | 14.00 (9.00, 20.00) | 3.60 (0.62, 6.26) | 8.00 (4.31, 13.00) |
| Mother | 370 (30.15%) | 2.00 (1.00, 3.00) | 4.16 (3.12, 8.00) | 22.00 (12.00, 35.75) | 2.50 (1.47, 4.00) | 10.29 (6.50, 15.00) | 257 (26.09%) | 2.00 (1.00, 2.00) | 5.00 (0.83, 11.00) | 13.50 (6.50, 21.17) | 2.79 (0.50, 5.67) | 8.00 (4.00, 12.00) |
| Child | 244 (19.89%) | 2.00 (1.00, 3.00) | 4.00 (0.62, 7.21) | 14.00 (2.67, 28.00) | 2.00 (0.39, 3.00) | 6.00 (1.63, 12.75) | 242 (24.57%) | 2.00 (1.25, 2.00) | 2.58 (0.82, 10.75) | 11.25 (3.19, 21.00) | 1.25 (0.50, 5.50) | 5.90 (1.94, 11.96) |
| Grandfather | 41 (3.34%) | 3.00 (2.00, 4.00) | 4.23 (3.12, 7.73) | 24.00 (10.00, 32.00) | 1.56 (0.62, 2.68) | 6.50 (3.40, 8.75) | 63 (6.40%) | 4.00 (2.50, 4.50) | 3.00 (0.94, 6.72) | 15.84 (7.00, 24.38) | 0.92 (0.41, 2.15) | 4.67 (2.14, 7.75) |
| Grandmother | 83 (6.76%) | 3.00 (2.00, 4.00) | 6.70 (2.03, 8.66) | 28.00 (14.00, 44.00) | 2.08 (0.62, 3.50) | 8.33 (3.92, 15.50) | 90 (9.14%) | 3.00 (2.00, 4.00) | 3.21 (0.64, 9.42) | 12.00 (4.12, 22.62) | 1.00 (0.30, 2.65) | 3.88 (1.73, 7.50) |
| Grandchild | 334 (27.22%) | 3.00 (2.00, 4.00) | 8.00 (4.32, 10.57) | 32.00 (21.00, 44.38) | 2.65 (1.98, 3.33) | 10.77 (7.00, 14.33) | 120 (12.18%) | 3.00 (2.00, 4.00) | 11.31 (2.34, 22.17) | 24.84 (12.38, 34.21) | 3.32 (0.75, 6.87) | 8.00 (3.60, 12.27) |
| Others | 15 (1.22%) | 2.00 (1.00, 4.00) | 4.78 (0.04, 7.41) | 16.00 (1.50, 20.29) | 1.32 (0.04, 1.78) | 2.94 (1.00, 6.53) | 12 (1.22%) | 2.00 (0.75, 3.00) | 0.56 (0.13, 1.04) | 10.50 (4.50, 25.25) | 0.17 (0.07, 0.41) | 5.25 (1.88, 7.44) |
| **Household size** |  |  |  |  |  |  |  |  |  |  |  |  |
| 2 | 216 (17.60%) | 1.00 (1.00, 1.00) | 4.00 (0.62, 4.00) | 12.00 (4.00, 20.00) | 4.00 (0.62, 4.00) | 12.00 (4.00, 20.00) | 230 (23.35%) | 1.00 (1.00, 1.00) | 1.00 (0.50, 8.33) | 9.00 (3.00, 14.75) | 1.00 (0.50, 8.33) | 9.00 (3.00, 14.75) |
| 3 | 316 (25.75%) | 2.00 (2.00, 2.00) | 4.16 (1.23, 8.00) | 19.00 (6.00, 30.00) | 2.31 (0.62, 4.00) | 10.00 (3.00, 15.00) | 286 (29.04%) | 2.00 (2.00, 2.00) | 3.00 (0.87, 9.65) | 12.00 (4.00, 19.00) | 1.94 (0.50, 5.16) | 6.39 (2.22, 11.00) |
| 4 | 324 (26.41%) | 3.00 (3.00, 3.00) | 7.12 (4.04, 9.00) | 26.50 (15.00, 44.00) | 2.50 (1.44, 3.50) | 10.00 (5.67, 15.54) | 237 (24.06%) | 3.00 (2.00, 3.00) | 7.08 (1.84, 14.55) | 17.00 (9.00, 26.00) | 2.67 (0.67, 5.88) | 7.00 (3.92, 10.83) |
| 5 | 168 (13.69%) | 4.00 (3.00, 4.00) | 8.04 (4.58, 9.62) | 31.50 (20.00, 42.00) | 2.16 (1.30, 2.83) | 8.50 (5.72, 11.67) | 128 (12.99%) | 4.00 (3.00, 4.00) | 9.71 (2.13, 17.75) | 22.38 (12.75, 34.51) | 2.75 (0.64, 5.19) | 6.18 (3.75, 10.50) |
| >5 | 203 (16.54%) | 5.00 (4.00, 5.00) | 10.23 (6.35, 12.66) | 38.00 (23.00, 52.00) | 2.31 (1.47, 2.75) | 7.75 (5.05, 10.94) | 104 (10.56%) | 5.00 (4.00, 5.00) | 6.79 (3.01, 17.75) | 23.12 (13.05, 37.47) | 1.84 (0.69, 3.66) | 5.28 (3.00, 9.00) |
| **Bedrooms in use** |  |  |  |  |  |  |  |  |  |  |  |  |
| 1 | 322 (26.24%) | 1.00 (1.00, 2.00) | 4.00 (4.00, 8.00) | 24.00 (13.00, 36.00) | 4.00 (2.50, 4.00) | 16.00 (11.00, 21.33) | — |  |  |  |  |  |
| 2 | 511 (41.65%) | 2.00 (2.00, 3.00) | 4.78 (2.50, 8.32) | 23.00 (9.00, 38.05) | 2.26 (1.17, 3.00) | 8.67 (4.50, 12.50) | — |  |  |  |  |  |
| 3 | 283 (23.06%) | 3.00 (2.00, 4.00) | 6.50 (2.56, 9.62) | 22.50 (8.50, 38.83) | 2.00 (0.83, 2.65) | 7.08 (3.23, 10.25) | — |  |  |  |  |  |
| 4 | 71 (5.79%) | 4.00 (3.00, 5.00) | 7.23 (2.98, 11.37) | 27.00 (15.00, 37.50) | 1.87 (1.02, 2.72) | 6.56 (3.92, 8.38) | — |  |  |  |  |  |
| >4 | 29 (2.36%) | 5.00 (3.00, 7.00) | 5.93 (4.08, 10.47) | 27.00 (18.00, 35.20) | 1.36 (0.67, 2.02) | 4.67 (3.60, 7.50) | — |  |  |  |  |  |
| Unknown | 11 (0.90%) | 2.00 (1.50, 2.50) | 0.90 (0.16, 8.00) | 2.90 (0.20, 37.50) | 0.18 (0.16, 2.83) | 2.00 (0.20, 14.18) | — |  |  |  |  |  |
| **Weather** |  |  |  |  |  |  |  |  |  |  |  |  |
| Sunny | 760 (61.94%) | 2.00 (1.00, 3.00) | 5.00 (4.00, 8.62) | 24.00 (10.00, 37.00) | 2.42 (1.30, 4.00) | 9.24 (4.50, 14.37) | — |  |  |  |  |  |
| Cloudy | 141 (11.49%) | 2.00 (2.00, 3.00) | 6.66 (4.00, 8.62) | 25.00 (16.00, 39.33) | 2.50 (1.68, 3.25) | 10.33 (6.75, 14.00) | — |  |  |  |  |  |
| Rainy | 271 (22.09%) | 2.00 (1.00, 3.00) | 4.24 (2.50, 8.00) | 23.00 (10.00, 38.00) | 2.19 (0.85, 3.23) | 9.33 (4.83, 14.00) | — |  |  |  |  |  |
| Varied | 50 (4.07%) | 2.00 (1.00, 3.00) | 4.16 (0.78, 8.83) | 18.00 (9.25, 38.00) | 2.05 (0.62, 3.00) | 8.00 (4.61, 13.25) | — |  |  |  |  |  |
| Unknown | 5 (0.41%) | 3.00 (2.00, 4.00) | 4.62 (1.23, 8.20) | 16.00 (14.00, 36.00) | 2.05 (0.62, 2.31) | 7.00 (5.33, 9.00) | — |  |  |  |  |  |
