# Supplementary material for: Role-specific household contact patterns in urban and rural China: Implications for influenza household transmission
Source: Infect Dis Model. 2026 Sep 2;12(1):154–69. doi: 10.1016/j.idm.2026.09.001 (PMC13634509; doi:10.1016/j.idm.2026.09.001)
Supplement: Supplementary file 2 [file mmc2.docx]

**Supplementary Figure 1. Distribution of household structure characteristics in Anhua and Kunming.** (A, B) Double-layer donut charts showing the distribution of family types (outer ring: One-Gen, Two-Gen, Three-Gen) and household sizes (inner ring: 2, 3, 4, 5, >5 persons); (C, D) Mean household size and number of participants by family type (error bars indicate standard deviations); (E, F) Scatter plots of household size versus number of participants (each point represents one household).

**Supplementary Figur**e **2. Frequency Distribution of Contact and Co-presence Duration**

**Supplementary Figure 3.** Forest plot of total contact duration and total co-presence duration in Anhua and Kunming.

**Supplementary Figure 4.** Age-specific contact duration and co-presence duration matrix across different generational structures in Anhua and Kunming.

**Supplementary Figure 5.** Role-specific contact and co-presence duration matrix stratified by household crowding levels in Anhua. Heatmaps illustrating the mean contact duration (left panels) and co-presence duration (right panels) among distinct family roles in Anhua, China. The data are categorized by generational structure and residential density: (A) Two-generation crowded households (< 0.6 bedrooms per capita); (B) Two-generation not crowded households (≥ 0.6 bedrooms per capita); (C) Three-generation crowded households (< 0.6 bedrooms per capita); and (D) Three-generation not crowded households (≥ 0.6 bedrooms per capita). Numeric values within each cell represent the mean duration in hours, estimated via 1,000 bootstrap resamplings. Color gradients denote the length of time, with darker shades corresponding to longer durations.

**Supplementary Figure 6.** Age-specific contact and co-presence duration matrix stratified by household crowding levels in Anhua.

**Supplementary Figure 7**. Sensitivity of the mean number of secondary cases to alternative truncation values for contact duration of “>4 hour” in Anhua.

**Supplementary Figure 8.** Validation of contact duration truncation using the empirical data in Kunming.

**Supplementary Figure 9.** Sensitivity of the mean number of secondary cases generated by each index role to the calibrated SAR of the father–mother pair.

 **Supplementary Figure 10.** Sensitivity of the mean number of secondary cases generated by each index role to the assumed infectious and latent periods. Panel A shows results for infectious periods of 3–7 days, and panel B shows results for latent periods of 1–3 days. Filled points denote the main-analysis values, while open points denote the alternative values evaluated in the sensitivity analysis. Lines are included as visual guides between evaluated values.

**Supplementary Figure 11**. Mean numbers of secondary cases generated by each index role across assumed relative susceptibility values for children and grandchildren.

**Supplementary Figure 12. Impact of** household crowding **on secondary case distributions in two-generation and three-generation households in Anhua.**

**Supplementary Figure 13.** Role-specific comparison of the mean number of secondary cases between crowded and non-crowded Anhua households under alternative crowding cutoffs

**Supplementary Figure 14**. Mean number of secondary cases in crowded and non-crowded Anhua households under alternative crowding cutoffs.

**Supplementary** **Figure 15. Transmission chains for different index case scenarios in households from Anhua and Kunming.** In all panels, the primary index case is highlighted with a larger, gold-colored node. Nodes represent individual household members (GF: Grandfather, GM: Grandmother, F: Father, M: Mother, C₁/C₂: Children, GC₁/GC₂: Grandchildren). **Edge thickness and corresponding percentage labels denote the transmission probability.**

**Supplementary Figure 16.** Transmission source analysis in two-generation and three-generation households from Anhua and Kunming. Stacked bar charts show the proportional attribution of within-household infection sources for each infected role, averaged across all possible index-case scenarios. (excluding scenarios where the target member themselves is the index case).

**Supplementary Figure 17.** Stability of vaccination-strategy rankings across assumed relative susceptibility values for children and grandchildren.

**Supplementary Figure 18.** Stability of vaccination-strategy rankings across calibrated SAR of the father-mother pair.

**Supplementary Figure 19.** Thresholds at which the optimal vaccination strategy changed when the external infection risks of the grandfather and grandmother were increased. Fold change is the sensitivity-analysis risk divided by the corresponding value used in the primary analysis; GCs denotes two grandchildren.

**Supplementary Figure 20.** Stability of optimal vaccination strategies under joint variation in grandchild and grandparent external infection risks in three-generation households.

**Supplementary Figure 21.** Stability of optimal vaccination strategies across 5,000 scenarios in which the external infection risks of all household roles were varied simultaneously. (A) The bars show the percentages of scenarios in which the optimal strategy of main analysis was retained (green) or an alternative strategy became optimal (other colors) at each dose level. (B) Each point represents one sampled scenario. The x-axis shows the fold change in the external infection risk of the role added to the alternative optimal strategy, whereas the y-axis shows that of the role it replaced.

**Supplementary Figure 22.** Robustness of optimal vaccination strategy rankings across varying levels of vaccine efficacy against susceptibility. Heatmaps displaying the top 5 most effective vaccination strategies for reducing secondary cases under different assumed levels of VEs (ranging from 40% to 90%). Results are stratified by household scenario and the number of available vaccine doses. The color gradient from green to red indicates the rank from 1st to 5th.

**Supplementary Figure 23.** Robustness of optimal vaccination strategy rankings across varying levels of vaccine efficacy against infectivity.

Heatmaps illustrating the rank of the top 5 vaccination strategies under different assumptions of vaccine efficacy against infectivity (ranging from 15% to 60%).

**Supplementary Figure 24.** Percentage reduction in mean secondary cases for vaccination strategies that were optimal in the main analysis.

**Supplementary Figure 25**. Impact of the father-mother pair SAR on the reduction in secondary cases under the top three vaccination strategies.

**Supplementary Figure 26.** Impact of vaccine efficacy against susceptibility on the reduction in secondary cases of top vaccination strategies.

Line charts illustrating how changes in *VE_S_* (ranging from 40% to 90%) affect the absolute reduction in secondary cases (%) for the top 3 vaccination strategies at each dose coverage level.

**Supplementary Figure 27.** Impact of vaccine efficacy against infectivity on the reduction in secondary cases of top vaccination strategies.

Line charts depicting the reduction in secondary cases (%) for the top 3 optimal vaccination strategies as *VE_I_* increases from 15% to 60%.
